# Scanner-specific optimisation of automated lesion segmentation in MS

**DOI:** 10.1016/j.nicl.2024.103680

**Published:** 2024-10-02

**Authors:** David R. van Nederpelt, Giuseppe Pontillo, Mar Barrantes-Cepas, Iman Brouwer, Eva M.M. Strijbis, Menno M. Schoonheim, Bastiaan Moraal, Bas Jasperse, Henk-Jan M.M. Mutsaerts, Joep Killestein, Frederik Barkhof, Joost P.A. Kuijer, Hugo Vrenken

**Affiliations:** a MS Center Amsterdam, Radiology and Nuclear Medicine, Vrije Universiteit Amsterdam, Amsterdam Neuroscience, Amsterdam UMC Location VUmc, Amsterdam, The Netherlands; b Queen Square Institute of Neurology and Centre for Medical Image Computing, University College London, UK; c MS Center Amsterdam, Anatomy and Neurosciences, Vrije Universiteit Amsterdam, Amsterdam Neuroscience, Amsterdam UMC Location VUmc, Amsterdam, The Netherlands; d MS Center Amsterdam, Neurology, Vrije Universiteit Amsterdam, Amsterdam Neuroscience, Amsterdam UMC Location VUmc, Amsterdam, The Netherlands

**Keywords:** Multiple sclerosis, Lesion segmentation, Reliability, Accuracy

## Abstract

**Background & Objective:**

Automatic lesion segmentation techniques on MRI scans of people with multiple sclerosis (pwMS) could support lesion detection and segmentation in trials and clinical practice. However, knowledge on their reliability across scanners is limited, hampering clinical implementation. The aim of this study was to investigate the within-scanner repeatability and between-scanner reproducibility of lesion segmentation tools in pwMS across three different scanners and examine their accuracy compared to manual segmentations with and without optimization.

**Methods:**

30 pwMS underwent a scan and rescan on three MRI scanners. GE Discovery MR750 (3.0 T), Siemens Sola (1.5 T) and Siemens Vida (3.0 T)). 3D-FLuid Attenuated Inversion Recovery (3D-FLAIR) and 3D T1-weighted scans were acquired on each scanner. Lesion segmentation involved preprocessing and automatic segmentation using the Lesion Segmentation Toolbox (LST) and nicMSlesions (nicMS) as well as manual segmentation. Both automated segmentation techniques were used with default settings, and with settings optimized to match manual segmentations for each *scanner specifically* and *combined* for the three scanners. LST settings were optimized by adjusting the threshold to improve the Dice similarity coefficient (DSC) for each scanner separately and a combined threshold for all scanners. For nicMS the last layers were retrained, once with the multi-scanner data to represent a combined optimization and once separately for each scanner for scanner specific optimization. Volumes and counts were extracted. DSC was calculated for accuracy, and reliability was assessed using intra-class correlation coefficients (ICC). Differences in DSC between software was tested with a repeated measures ANOVA and when appropriate post-hoc paired t-tests using Bonferroni correction.

**Results:**

Scanner-specific optimization significantly improved DSC for LST compared to default and combined settings, except for the GE scanner. NicMS showed significantly higher DSC for both the scanner-specific and combined optimization than default. Within-scanner repeatability was excellent (ICC>0.9) for volume and counts. Between-scanner ICC for volume between Vida and Sola was higher (0.94–0.99) than between GE MR750 and Vida or Sola (0.18–0.93), with improved ICCs for nicMS scanner-specific (0.87–0.93) compared to others (0.18–0.79). This was not present for Sola vs. Vida where all ICCs were excellent (>0.94).

**Conclusion:**

Scanner-specific optimization strategies proved effective in mitigating inter-scanner variability, addressing the issue of insufficient reproducibility and accuracy found with default settings.

## Highlights

• Accuracy and reliability of lesion segmentation tools in MS were investigated.
• Default settings were insufficient for clinical implementation.
• Domain adaptation strategies improve accuracy and reliability.

## 1 Introduction

Multiple sclerosis (MS) is characterized by the distinctive combination of focal demyelination and neurodegeneration within the central nervous system (CNS) (Filippi et al., 2018). Magnetic resonance imaging (MRI) is an invaluable non-invasive tool for visualizing brain lesions in MS. The high sensitivity of MRI for lesions allows for qualitative and quantitative assessments of lesion burden, primarily on T2-weighted images (Wattjes et al., 2021). MS lesions can take diverse shapes but are mainly focal and occur in various locations, including periventricular, (juxta)cortical, infratentorial brain regions, and the spinal cord, all of which are incorporated into the revised 2017 McDonald criteria as part of the dissemination in space criterion (Thompson et al., 2018).

Automatic lesion segmentation techniques could serve as a valuable aid for both lesion detection and segmentation in research, clinical trials and are starting to make their way into the clinical neuroradiology workflow, although some hurdles still need to be overcome (Spagnolo et al., 2023). These tools have shown to be promising candidates to alleviate the time burden, mitigate inter-observer variability and provide quantitative information on lesion volumes (Weeda et al., 2019). The latter is relevant to use in a clinical context for, among others, disease progression predictions which has shown to be more promising than lesion count alone (Oship et al., 2022).

The majority of current studies assessing automatic lesion segmentations have focused on the accuracy of these tools (Weeda et al., 2019, Sadeghibakhi et al., 2022). However, little research has been dedicated towards the reliability of these tools, which hampers clinical implementation. The present study investigated the within-scanner repeatability and between-scanner reproducibility, as defined previously (van Nederpelt et al., 2023), of automated lesion segmentation tools on three different MR scanners with different field strengths in MS patients with different degrees of pathology. Between-scanner reproducibility included differences in both hardware and clinical scan protocols. Furthermore, we investigated whether local optimization schemes for automatic segmentation tools could improve these metrics.

## 2 Materials and methods

### 2.1 Participants

In this study, 30 people with MS (pwMS), aged between 18 and 70 years, were scanned between February and June 2022, as part of the “accurate multiple sclerosis atrophy measurement system” (AMS2) dataset (van Nederpelt et al., 2024). This included 22 relapsing-remitting MS (RRMS), 2 secondary progressive MS (SPMS) and 6 primary progressive MS (PPMS) participants, diagnosed according to the revised 2017 McDonald criteria (Thompson et al., 2018). Exclusion criteria were past or current clinically relevant non-MS neurological, psychiatric or (auto)immune disorder(s), as well as contraindications to undergo MRI examination. The participants were scanned twice (scan and rescan), to assess within-scanner repeatability (or precision), on three MR scanners to assess between-scanner reproducibility, in the same center. Between the scan (run 1) and rescan (run 2), the participants got off the scanner bed and walked a few steps before repositioning. All the different MR examinations were performed on the same day within 4 hours, to minimize time of day effects. Participants did not consume coffee in between scans, but could maintain normal hydration status by consuming tea and water or visiting the bathroom as needed. The local ethical committee approved the study protocol (NL75420.029.20), and written informed consent was obtained from all individuals, according to the Declaration of Helsinki. During their visit, the expanded disability status scale (EDSS) score was assessed by means of a questionnaire (Lechner-Scott et al., 2003).

### 2.2 MRI protocol

The scans were performed on three MRI scanners: 1) 3.0 T GE Discovery MR750 (General Electric Healthcare, Milwaukee, USA), 2) 1.5 T Siemens Sola and 3) 3.0 T Siemens Vida (Siemens Healthineers, Erlangen, Germany). All exams were performed by the same trained PhD-candidate (DvN). The imaging protocol included a 3D T1-weighted (T1w) and a 3D FLuid Attenuated Inversion Recovery (FLAIR) scan using acquisition protocols (Table 1). Because the aim of this study is to investigate how lesion segmentation is affected by scanner differences in a clinical setting, we did not standardize echo time (TE), repetition time (TR) and inversion time (TI) between scanners. In the current study, we will use the term “between-scanner” to encompass both the differences arising from using different scanners, including differences in acquisition protocols. For all acquisitions, the vendor-specific 3D correction for geometric distortion from non-linearity of gradient coils was applied on the scanner during image reconstruction. Images were visually checked for artifacts (e.g., movement) at the time of scanning and reacquired immediately if deemed necessary.

**Table 1:** MRI acquisition parameters1.

**Scanner**	**Pulse sequence**	**resolution (mm^3^)**	**TR (ms)**	**TE (ms)**	**TI (ms)**	**FA (°)**		**Field Strength (T)**
**GE Discovery MR750**	IR-FSPGR	1.0 x 1.0 × 1.0	8.2	3.2	450	12		3
**Siemens SOLA**	MPRAGE	1.0 x 1.0 × 1.0	2300	2.6	900	8		1.5
**Siemens Vida**	MPRAGE	1.0 x 1.0 × 1.0	2300	2.3	900	8		3
**GE Discovery MR750**	FLAIR	0.98 x 0.98 x 1.2	8000	129.1	2340	−		3
**Siemens SOLA**	FLAIR w/ T2-prep	1.0 x 1.0 x 1.4	5000	388	1600	−		1.5
**Siemens Vida**	FLAIR w/ T2-prep	1.0 × 1.0 x 1.0	5000	386	1650	−		3

1 IR-FSPGR=Inversion Recovery Fast SPoiled GRadient Echo, MPRAGE=Magnetization Prepared RApid Gradient Echo, FLAIR=FLuid Attenuated Inversion Recovery, TR=Repetition Time, TE=Echo Time, TI=Inversion Time, FA=Flip Angle.

### 2.3 Lesion segmentation

#### 2.3.1 Preprocessing

Prior to lesion segmentation, both FLAIR and T1w scans were bias field corrected using the N4 algorithm from the advanced normalization tools (ANTs) software package (Tustison et al., 2010, Avants et al., 2009). The T1w scan was linearly registered to the FLAIR with FMRIB's Linear Image Registration Tool (FLIRT), using 6 degrees of freedom (DOF) and default parameters. Subsequently, the FLAIR-derived brain mask, created using the HD-brain extraction tool (HD-BET), was applied to both FLAIR and T1w scans to ensure uniformity in BET results (Isensee et al., 2019, Jenkinson and Smith, 2001).

#### 2.3.2 Manual segmentation and lesion location classification

##### 2.3.2.1 Manual segmentation

**Fig. 1:**
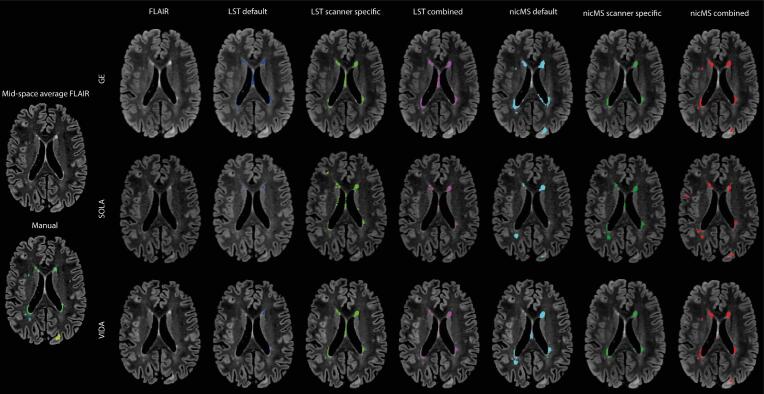
Example of lesion segmentations, FLAIR images and the mid-space average FLAIR. Red arrows indicate which FLAIR images were used to create the mid-space average. For the manual segmentation, green indicates periventricular lesions, lightblue indicates deep lesions, and yellow indicates (juxta)cortical lesions. For the automated segmentation techniques: dark blue = LST default, light green = LST scanner specific, pink = LST combined, light blue = nicMS default, green = nicMS scanner specific, red = nicMS combined. (For interpretation of the references to colour in this figure legend, the reader is referred to the web version of this article.)

For manual lesion segmentation, a mid-space average image was created using the brain-extracted and N4 bias field-corrected FLAIR scans obtained from the first run of the three different scanners. The subject-specific mid-space average was constructed using the ANTs “antsMultivariateTemplateConstruction2.sh” with default options, apart from rigid-body registration of the three scans to an unbiased mid-space and averaging across scans (See Fig. 1) (Avants et al., 2009). This procedure was followed to ensure unity between the different lesion masks in the native space.

Two raters (MB, DvN) manually delineated lesions on the mid-space image using FSLeyes separately (10 and 20 images, respectively). We defined lesions as hyper-intense regions with or without a hypo-intense core on the FLAIR compared to the surrounding tissue with a size of at least 5 voxels (5 mm^3^). The raters had access to the T1w image registered to the FLAIR mid-space image as a reference. Additionally, the raters rated three participants twice to calculate the intra-rater agreement. Afterwards, an experienced MS neuroradiologist (GP, experience > 10 years) reviewed every segmentation (30 in total), and lesion masks were adjusted accordingly.

##### 2.3.2.2 Lesion classification

For the automatic lesion location classification, Synthseg (v2.0) was run on the mid-space average FLAIR image and cortical, lateral ventricle, brainstem and cerebellum masks were obtained (Billot et al., 2023). First, lesions were classified as periventricular if lesions overlapped with the lateral ventricles mask which was dilated with a spherical kernel of 1 mm. Second, lesions were classified as (juxta)cortical if lesions had at least 5 voxels overlapping with the cortical mask. Third, lesions were classified as infratentorial if they overlapped with the cerebellum and/or brainstem mask. Fourth, all other lesions were classified as deep WM, based on an eroded (spherical kernel 2 mm) cerebellar WM mask, or as “other”. Because less than 3 % was classified as “other” and most were in the deep WM category, these two categories were merged into a single category “deep”. During the review of the segmentations, the neuroradiologist also checked for the lesion classifications and lesion location classifications were changed accordingly.

The final manual lesion mask was registered back to all three native spaces (the first run of each scanner). Here the inverse of the registration matrix of the mid-space average construction and AntsApplyTransforms from the ANTs toolbox with nearest neighbor interpolation was applied.

#### 2.3.3 Automatic segmentation

Two segmentation tools were selected for evaluation, as they were the most promising candidates with respect to accuracy based on a previous study (Weeda et al., 2019). These were the Lesion Prediction Algorithm (LPA) as implemented in the Lesion Segmentation Toolbox (LST) toolbox version 3.0.0 (www.statistical-modelling.de/lst.html) (Schmidt and Wink, 2017) and nicMS lesions version 0.2 (https://github.com/sergivalverde/nicMSlesions) (Valverde et al., 2019). For both software packages, the lesion volume and counts were obtained using the fslstats and fsl-cluster tools from the FSL library version 6.0.6.5 (Jenkinson et al., 2012).

##### 2.3.3.1 LST

LST-LPA is an open-source toolbox integrated into the Statistical Parametric Mapping (SPM) toolbox. Initially developed for MS lesion segmentation, LPA was trained using a logistic regression model based on data from 53 pwMS with severe lesion patterns (Schmidt, 2017). The output of LST is a lesion probability map. To evaluate whether optimization of the threshold for probability influences the accuracy and reliability, three thresholds were applied. First, the default threshold of 0.5 was applied, termed: “LST *default”.* Then, the threshold was adjusted for each scanner specifically as previously described (Weeda et al., 2019), resulting in an optimized threshold for GE, an optimized threshold for SOLA and an optimized threshold for the VIDA FLAIR scans termed: “LST *scanner specific*”. Briefly, one participant, the one with the median lesion volume, was selected from the dataset. The threshold was increased with a 0.05 step size from 0.1 to 0.9. The threshold that yielded the highest dice similarity coefficient (DSC) for that participant was selected. Lastly, a combined threshold for all three scanners was defined, which was based on the highest DSC for 3 participants that were closest to the median lesion volume of the group: “LST *combined scanners*”. Participants selected for optimization were not used for performance evaluation.

##### 2.3.3.2 nicMSlesions

NicMSlesions (nicMS) is an open source supervised deep learning approach based on a convolutional neural network (CNN) to achieve precise segmentation of white matter (WM) lesions. NicMS can be re-trained with a limited set of annotated images (Valverde et al., 2019). At first, we applied the default network “baseline 2ch” for the automatic segmentation. Secondly, as retraining with one subject significantly improved the segmentation (Weeda et al., 2019); nicMS was retrained in the following situations;

a) A scanner-specific network. Here, the 2 last layers of nicMS were retrained three times separately using the manual lesion mask of one participant, with the median lesion volume, registered back to the native FLAIR space of each scanner. In this manner, three specific retrained versions were created, namely, a GE, SOLA and VIDA version. These versions are referred to as nicMS *scanner specific*. The participant was not used for performance evaluation.
b) A combined network for all the scanners. For this, we selected lesion masks of 3 participants that were closest to the median lesion volume of the group, based on the manual segmentation. We randomly paired participants with scanners and registered the manual lesion mask to the corresponding native FLAIR space, resulting in one manual lesion mask in the native FLAIR space of the GE, one in the native FLAIR space of the SOLA and the last for the VIDA native FLAIR space. These three masks were used to retrain one network. This network version is called nicMS *combined scanners.* The three participants were excluded from the performance evaluation.

The participants used for optimalisation were the same for both LST and nicMS. Detailed demographic information is provided in the Supplementary Table 1.

For both manual and automatic segmentations, a minimum cluster size of 5 voxels was applied to ensure unity between the minimum lesion size, using the fsl-cluster tool (Jenkinson et al., 2012), with maximum connectivity of 26 (faces, edges and corners). For the manual segmentation, this was done in the mid-space image before registration to the native FLAIR space. A potentially higher reproducibility for lesions was tested with cluster sizes of 10 and 20 voxels.

### 2.4 Statistical analyses

All statistical analysis was performed using R Statistical Software (version 4.1.1; R Foundation for Statistical Computing, Vienna, Austria).

#### 2.4.1 Accuracy

Accuracy was assessed using the DSC in the native FLAIR space of each scanner. This was extracted from images for the first run only. Differences in DSC were tested using a repeated measures analysis of variance (ANOVA) with paired *t*-test for post hoc analyses. Other measures included the lesion-wise true positive rate (TPR), false positive rate (FPR), and false negative rate (FNR) for lesion detection. The distribution of TPR, FPR and FNR over typical lesion locations was calculated as well. Rates were calculated with reference to the total lesion count derived from the manual segmentations.

#### 2.4.2 Reliability

The reliability part of this study used the same analysis pipeline as previously described (van Nederpelt et al., 2023). The following analyses were conducted for both lesion volume and count: 1) the intra-class correlation coefficient (ICC) with a 95 % confidence interval (CI) for absolute agreement within scanner (ICC-AA) for the output of run 1 and run 2. 2) the ICC for consistency between scanners (ICC-C), based on run 1 of all pairwise scanner combinations. 3) reproducibility evaluations of volume and counts involved a repeated measures ANOVA and a Friedman test, respectively. Classification of ICC values was performed according to proposed reporting guidelines (Koo and Li, 2016). In cases where Mauchly’s test for sphericity was violated, a Greenhouse-Geisser correction was applied. Subsequent post hoc examinations employed pairwise t-tests or Wilcoxon signed-rank tests, when appropriate. 4) to compute the standard error of measurements (SEM) and the smallest detectable change (SDC), a variance component analysis (VCA) was performed for both within-scanner (SEM_within_, SDC_within,_) and between-scanner (SEM_between_, SDC_between_) measurements (de Vet et al., 2011, Mokkink et al., 2023). These measures are presented as percentages of the mean lesion volume or count across all subjects. Reported *p*-values are Bonferroni corrected for multiple comparisons. The threshold for statistical significance was set at α = 0.05.

## 3 Results

### 3.1 Demographics and training time

The demographics of the pwMS are detailed in Table 2. The cohort included 10 males (33 %), with an average age of 44.4 ± 11.7 years, ranging from 21.7 to 61.9 years. The average disease duration was 7.4 ± 6.4 years and the mean EDSS score was 3.3 ± 1.9, ranging from 0 to 6.5. Lesion volume averaged 5.9 ml, with a wide range from 1.7 to 51.2 ml. For nicMS the training time was 48.1 min for the combined network. For the GE scanner specific network this was 19.28 min, and 13.72 and 19.95 min for the Sola and Vida, respectively.

**Table 2:** Demographics and clinical characteristics^2^. ^a^ Mean, ^b^ Mean since symptom onset, ^c^Median based on manual segmentation.

	MS (n = 30)
**Demographics**	
Subject type (RRMS/SPMS/PPMS)	22/2/6
Male, n (%)	10 (33 %)
Age^a^, y (range)	44.4 ± 11.7 (21.7–61.9)
Disease duration^b^, y (range)	7.4 ± 6.4 (0.7–23.8)
**Clinical characteristics**	
EDSS^a^ (range)	3.3 ± 1.9 (0–6.5)
Lesion^c^ volume, ml (range)	5.9 (1.7–51.2)
**Disease-modifying treatment**	
(none/TEC/GIL/COP/TYS/OCR/AUB)	81/1/3/1/9/1
**Non-disease modifying drug**	
(FAM)	6

2 TEC=dimethyl fumarate (Tecifidera); GIL=fingolimod (Gilenya); COP=glatiramer acetate (Copaxone); TYS=Natalizumab (Tysabri); OCR=Ocrelizumab (Ocrevus); AUB=Teriflunomide (Aubagio); FAM=fampridine (Fampyra).

### 3.2 Accuracy

#### 3.2.1 Manual segmentation

The repeatability of the manual segmentations was spatially good, and volumetrically excellent. The ICC for absolute agreement of the volumes was 0.93, and the mean DSC was 0.73 ± 0.06. From the automated lesion location method, 93 % was correctly classified (total manual segmented lesion count = 1666). The most common corrections made by the neuroradiologist were from (juxta)cortical to deep (2.7 %) and from deep to periventricular (2.04 %).

#### 3.2.2 Automated segmentation

**Fig. 2:**
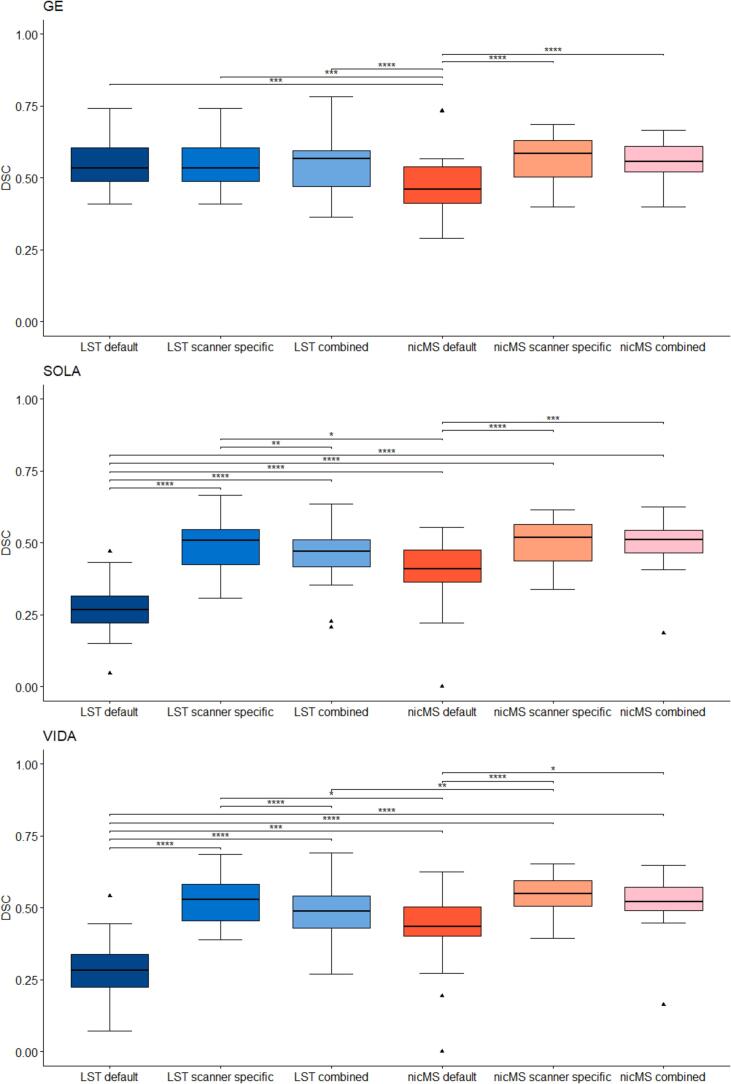
Boxplot of Dice similiarity coefficient (DSC) values for each scanner separately. Top = GE, middle = SOLA bottom = VIDA.

**Fig. 3:**
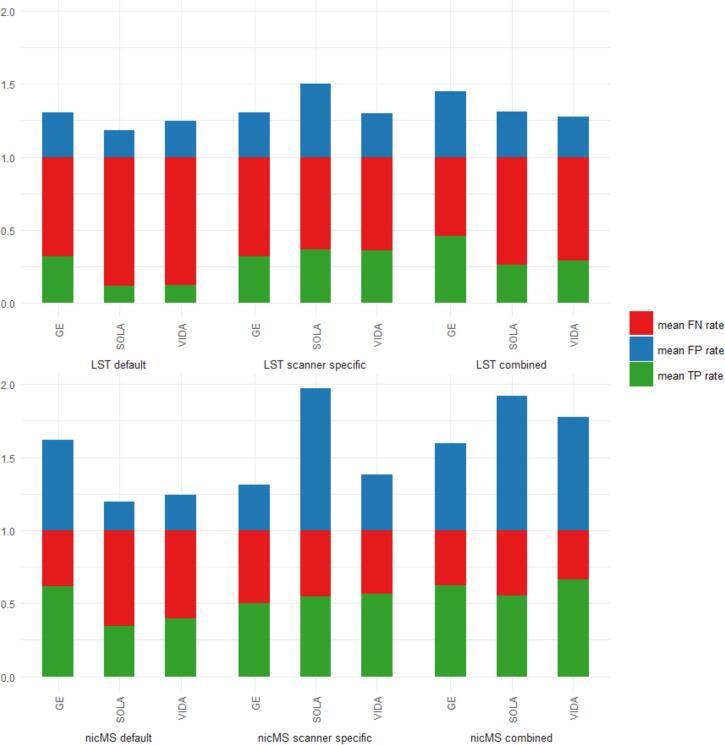
Stacked bar graph of mean true positive (TP), false positive (FP) and false negative (FN) rates for all scanners and segmentation methods combined. Rates were calculated based on individual manual lesion counts.

For both LST and nicMS, the DSC was different between the software and optimization procedures, as the main effect of the repeated measures ANOVA was significant for every scanner. The DSC improved significantly (*p* < 0.05*)* for the scanner-specific (range: 0.49–0.56) and combined optimization (range: 0.46–0.56) compared to default DSCs (range: 0.27–0.47), except for LST on GE scans (see Fig. 2). A detailed overview of the DSC values is provided in Table 3. For the Sola and Vida, the DSC for LST-scanner-specific optimization (Sola: 0.49, Vida 0.53) was significantly higher than LST-combined optimization (Sola: 0.46, Vida 0.47). The DSC for nicMS was significantly higher than LST default for Sola and Vida but for the GE, conversely, LST default outperformed nicMS. There was no difference between the DSC of nicMS scanner-specific and nicMS combined (*p* > 0.05). In the supplementary Figs. 1-6, all volumes and counts of automated segmentations are plotted against the manual segmentations. The TPR, FPR and FNR are depicted in Fig. 3. Increased TPR and lower FNR are observed for nicMS, although this was not statistically tested. The FPR for nicMS scanner specific was visually increased compared to nicMS combined for the GE and Vida while this was reversely true for the Sola. This was also seen for LST. Supplementary Figs. 7-9 show the share of each lesion location is depicted with respect to the TPR, FNR and FPR. It can be observed that the highest fraction of the TP lesions for LST are found in the periventricular area whereas for nicMS the highest TP fraction was observed for deep lesions. For LST there were almost no TP infratentorial lesions. No visible differences between scanners can be observed. Interestingly, most FN lesions were in the deep category for both LST and nicMS and did not differ between scanners (see Supplementary Fig. 8). The location for FP lesions differed between scanners and segmentation tool (Supplementary Fig. 9).

**Table 3:** DSC^3^ values for LST and nicMS.

	LST default	LST scanner specific	LST combined	nicMS default	nicMS scanner specific	nicMS combined
GE	0.55 ± 0.09	0.55 ± 0.09	0.55 ± 0.09	0.47 ± 0.11	0.56 ± 0.08	0.56 ± 0.08
Sola	0.27 ± 0.11	0.49 ± 0.09	0.46 ± 0.10	0.40 ± 0.11	0.50 ± 0.08	0.49 ± 0.08
Vida	0.28 ± 0.10	0.53 ± 0.08	0.47 ± 0.09	0.43 ± 0.12	0.54 ± 0.07	0.52 ± 0.09

3 DSC=Dice score coefficient.

### 3.3 Reliability & precision

#### 3.3.1 Within-scanner

**Fig. 4:**
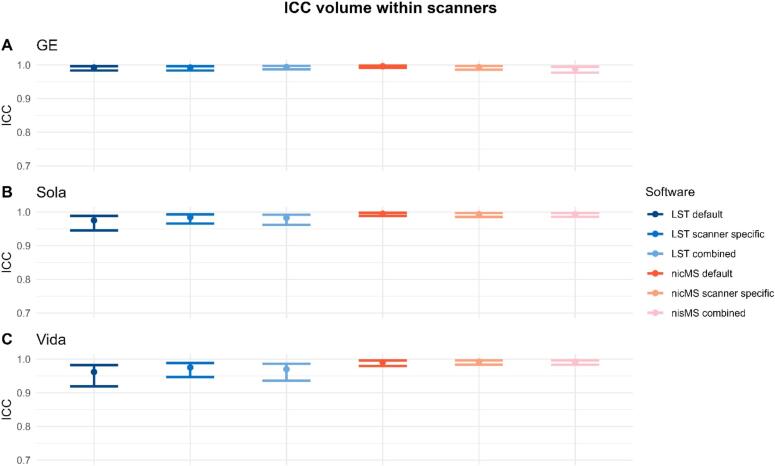
The Intra-class correlation coefficient (ICC) for absolute agreement for lesion volume within scanners. A is the GE scanner, B the Sola and C the Vida. The error bars indicate the 95% confidence intervals.

**Fig. 5:**
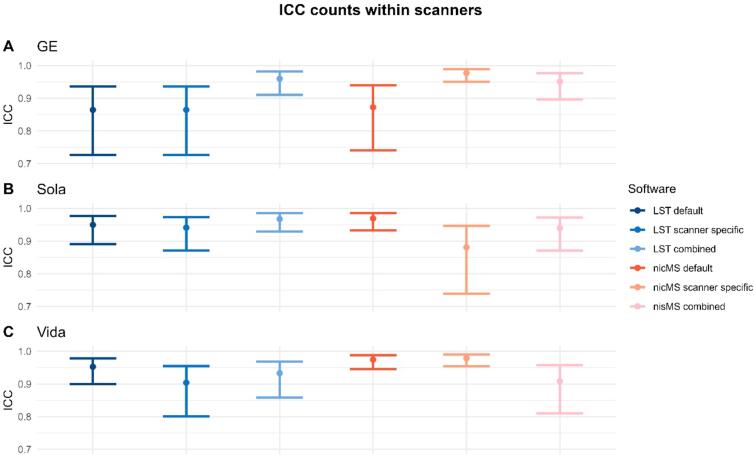
The Intra-class correlation coefficient for absolute agreement within scanners for the lesion count. The error bars are the 95% confidence intervals.

The ICC-AA for within scanner volumes was excellent (>0.9) and is depicted in Fig. 4, the 95 % CI were slightly wider for LST for the Vida and Sola. The data for counts and volumes is additionally depicted in the Supplementary Materials (Supplementary Figures 9–15), where each run is plotted against the second run of the same scanner. Similar observations were found for the counts (Fig. 5). However, for the GE (LST default, LST scanner specific and nicMS default) and Sola (nicMS scanner specific), the ICC was good (>0.85) instead of excellent. SEM and SDC values are detailed in Table 4. SEM and SDC values were slightly lower for nicMS for both volumes and counts.

**Table 4:** SEM and SDC^4^ within the scanner for the volumes and counts. The values are presented as percentage of the mean lesion volume and counts, as obtained for the software (LST or nicMS) used.

	SEM volume	SDC volume	SEM count	SDC count
LST				
*default*	14.19 %	39.32 %	15.64 %	43.36 %
*scanner specific*	11.18 %	31.00 %	13.06 %	36.21 %
*combined*	10.37 %	28.74 %	9.49 %	26.30 %
nicMS				
*default*	6.68 %	18.50 %	10.67 %	29.57 %
*scanner specific*	7.47 %	20.70 %	9.02 %	25.00 %
*combined network*	7.29 %	20.19 %	11.35 %	31.47 %

4 SEM=Standard error of measurement; SDC=smallest detectable change.

#### 3.3.2 Between-scanner

**Fig. 6:**
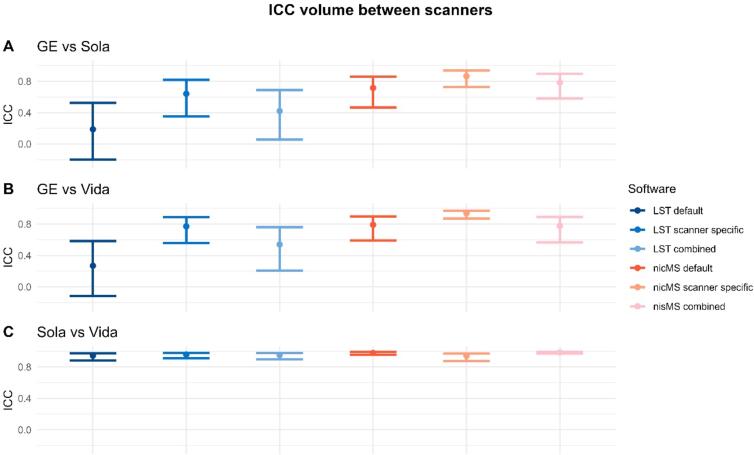
The Intra-class correlation coefficient for consistency between scanners for the lesion volume. The error bars are the 95% confidence intervals.

**Fig. 7:**
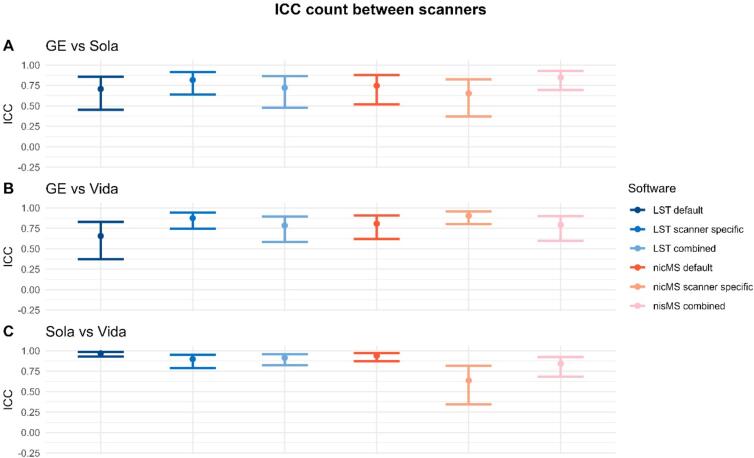
The Intra-class correlation coefficient for consistency between scanners for the lesion count. The error bars are the 95% confidence intervals.

**Fig. 8:**
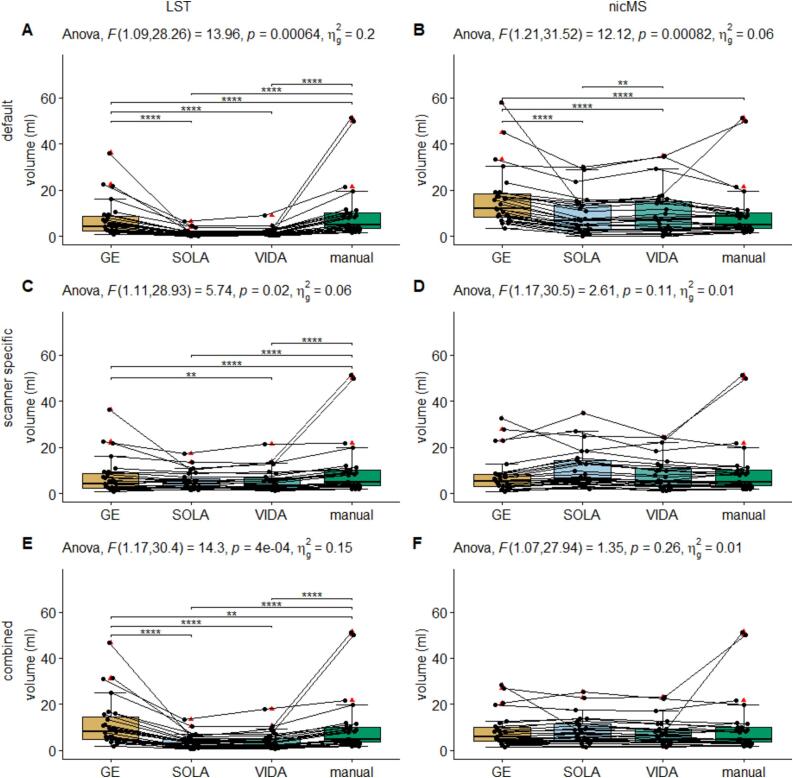
Boxplot of lesion volumes for each scanner and the manual segmentation. A=LST default, B=nicMS default, C=LST scanner specific, D=nicMS scanner specific, E=LST combined, F=nicMS combined. Lines indicate the same subject for the different scanners.

Between-scanner ICC-C for volumes varied between software and scanner comparison (see Fig. 6). For Sola vs Vida, ICCs were excellent (>0.94) for both LST and nicMS. However, this was not true for both Siemens scanners vs the GE scanner. The scanner-specific optimization led to a significant (non-overlapping CIs) increase in ICC for nicMS *scanner specific* vs. LST *default* and LST *combined* and for LST *scanner-specific* vs LST *default* for the GE vs Vida. A detailed overview of data for counts and volumes is additionally depicted in the Supplementary Materials (Supplementary Figures 17–22), where each first run of the scanner is plotted against the first run of the other scanners. ICC for counts was good to excellent for all scanners and tools (Fig. 7). Lesion size did not influence the ICC values for volume as ICCs were comparable between clusters of 5, 10 and 20 voxels (see Supplementary Figure 23). A minimum of threefold increase in SEM and SDC was observed (Table 5) compared to within-scanners, and the lowest values were found for *scanner-specific* optimization for volumes and *combined* optimization for counts, which was true for both nicMS and LST. Fig. 8 depicts the boxplots for volumes of every scanner and segmentation method. For every software variant, significant differences (p < 0.02) were found between the scanners but also between scanners and manual volumes. This was not present for nicMS *scanner specific* (p = 0.11) and nicMS *combined* (p = 0.26).

**Table 5:** SEM and SDC^5^ between scanners for the volumes and counts. The values are presented as percentage of the mean lesion volume and counts of the specific software.

	SEM volume	SDC volume	SEM count	SDC count
LST				
*default*	154.24 %	427.54 %	34.37 %	95.26 %
*scanner specific*	51.56 %	142.91 %	24.41 %	67.65 %
*combined*	100.06 %	277.34 %	21.06 %	58.37 %
nicMS				
*default*	50.75 %	140.67 %	33.84 %	93.81 %
*scanner specific*	31.00 %	85.93 %	30.83 %	85.47 %
*combined network*	35.02 %	97.06 %	23.23 %	64.39 %

5 SEM=Standard error of measurement; SDC=smallest detectable change.

## 4 Discussion

In this study, the accuracy, reliability and precision of two distinct lesion segmentation tools for MS, namely LST and nicMS, were assessed in a unique same-day scan-rescan study using three different MRI scanners with different field strengths. Both tools were optimized for each scanner separately and for the three scanners combined. NicMS *scanner-specific* optimization outperformed both the *default* and combined optimization, except for the SEM and SDC where a slight increase was found with respect to lesion count. Moreover, there were no significant differences between scanners for the AI-based nicMS algorithm for *scanner-specific* and *combined* optimization with respect to volume, which were present for LST. LST *scanner-specific* optimization also improved the SEM and SDC for lesion count and volume. However, for LST, the optimum threshold for the scanner *specific-optimization* on GE was the identical to the *default* threshold (both 0.5).

The DSC for scanner-specific optimized tools (0.52–0.54) presented in this study are comparable to manual inter-rater variability and other studies which investigate automatic lesion segmentation accuracy. Although these DSC values may seem low compared to whole brain segmentation tasks, it is important to recognize that the maximum accuracy achievable by automatic tools is inherently limited by inter-rater variability, which serves as the benchmark for comparison to a gold standard. Additionally, there is a relationship between the region of interest (ROI) size and DSC values, where a smaller ROI (e.g., lesions) results in a lower DSC value than a larger ROI (e.g., a whole brain mask) with a similar deviation in contour. The comparable results to rater variability suggest that the automatic lesion segmentation tools are sufficiently accurate for clinical application (Weeda et al., 2019, Valverde et al., 2019, Commowick et al., 2021). In addition, lesion masks obtained through automatic segmentation can be helpful for accurate brain volume quantification in pwMS (González-Villà et al., 2017). Not to mention, they could aid in lesion detection as radiologists currently perform visual identification of lesions on T2-weighted sequences, a time-consuming process and susceptible to high variability among different radiologists (Bozsik et al., 2022). It must be noted that SEM and SDC values for lesion count here are provided as a percentage of the mean lesion count and that for the subject with the lowest lesion count (14 lesions), optimized tools offer a precision of 1.3 lesions which is relevant for demonstrating evidence of disease activity (Kappos et al., 2016).

In this study, segmentation of MS lesions was optimized for each individual scanner using only one MRI exam. The relatively short time investment of one manual segmentation of one subject could, therefore, be a worthwhile effort to be able to obtain reliable and accurate automatic lesion masks between different scanners or potentially even between different centers, but this was not tested in this study. Moreover, the improvement of optimization with just one individual lesion mask has already previously been shown to improve accuracy, affirming our results (Weeda et al., 2019). However, incorporating multiple subjects with varying lesion loads (small, medium, and high) could further enhance the robustness of the model.

Within-scanner reliability was high, however, the time between repeated scans was short as this was a scan-rescan type of approach. It could be argued that this overestimates precision because long-term scanner hardware variability is not included. It should be noted that all exam-specific tuning and calibration (such as resonance frequency, B0 shim and transmitter amplitude) are fully repeated for the rescan. Variability from long-term fluctuations of B0 field homogeneity and gradient amplitude calibration are however not included. Additionally, repositioning effects may also be slightly larger over a longer time span, because the operator and the patient may inadvertently tend to reproduce head positioning better on such short term.

Between vendors, lesion masks of the Siemens scanners seemed to be more similar when compared to the GE (3 T) even though there is a difference in field strength between the Sola (1.5 T) and Vida (3 T). This result is slightly unexpected as several studies have indicated effects of field strength on lesion detection and segmentation (Sicotte et al., 2003, Hagens et al., 2018). One of these studies indicated that 15 % more T2 lesions were found at 3 T then at 1.5 T, but this was assessed visually by radiologists (Hagens et al., 2018), which is slightly different from the current study. A possible explanation could be that an identical implementation of the 3D FLAIR pulse sequence was used on the Siemens scanners, while the GE implementation visually appears to yield a slightly different contrast. This discrepancy may arise due to subtle variations in the evolution of the variable flip angle or the FSE/TSE echo train of the CUBE and SPACE sequences. We used a T2 magnetization preparation on the Siemens scanners module before the inversion pulse. This reduces T1-weighting for GM and WM, allowing a shorter TR. This preparation was not available on the GE scanner. Moreover, there was an increased FPR for the Sola scanner for scanner-specific optimization. This could possibly result from manual lesion masks being created in the combined mid-space average space, where the resulting FLAIR images are likely to have an increased signal-to-noise ratio compared to the original FLAIR images in the native space. This is even more true for the Sola scanner, which has a lower field strength compared to the other two scanners (1.5 T vs. 3 T). Again, it is well known that field strength influences the detection and segmentation of lesions and more lesions as well as lesion volume are found on 3 T compared to 1.5 T (Sicotte et al., 2003).

In the context of multi-center studies, an important consideration is whether to include scanner (center) effects in the statistical model, as scanner variability can introduce bias or affect the robustness of the results (van Nederpelt et al., 2023, Biberacher et al., 2016). Several statistical harmonization techniques have been proposed for this (Fortin et al., 2018, Pomponio et al., 2020). However, the proposed domain adaptation technique here could help mitigate scanner effects by aligning the data distributions across different scanners, potentially reducing the need for explicit scanner correction as volume measurements do not statistically differ for nicMS *scanner*-*specific* optimization and nicMS *combined*. However, correcting for center effects can still be beneficial, as patient selection criteria may vary across centers, introducing additional variability. In our study, the focus was on clinical application within the same patient context where such statistical models are not applicable, but future work should consider these broader statistical modelling questions.

Furthermore, it is noteworthy that nicMS currently needs the T1w images as additional input, which may limit its applicability in the clinical routine where T1w images are not routinely acquired. In contrast, LST-LPA requires only FLAIR images, rendering it more accessible for standard clinical use. Although our study utilized both T1w and FLAIR images for consistency, the broader clinical implications of these differences merit consideration. Future studies should focus on optimizing FLAIR-only segmentation tools. Next to software optimization, other strategies for the harmonization of lesion segmentations could be employed. For example, in a recent study, it was shown that using image harmonization of same-subject between-scanner T1w and FLAIR scans, the intra-rater agreement improved both for spatial distribution and for global and per-lesion volume between the scanners (Carass et al., 2024). The problem is that this image harmonization strategy was trained on paired data. Alternative harmonization strategies with unpaired data could be used for future endeavours (Zuo et al., 2023). For clinical introduction of the proposed methods future endeavors should follow the Quantitative neuroimaging framework as previously proposed (Goodkin et al., 2019).

### 4.1 Limitations

This study has several limitations. Firstly, as the manually segmented lesions were registered from the mid-space average space to the original FLAIR space, small lesion masks may have shifted slightly. However, the minimum lesion size of 5 voxels limited the impact. Secondly, the combined mid-space average of all three scanners improved the image quality both in terms of contrast- and signal-to-noise. This could have resulted in better-defined lesions compared to single scanner FLAIR images. Lastly, although this study was a scan-rescan study using different MRI scanners with different field strengths, it was performed in a single center with one dedicated person in charge of image acquisition.

## 5 Conclusion

This study showed a higher within-scanner repeatability than between-scanner reproducibility for lesion segmentations in MS. Using default settings the between-scanner accuracy and reproducibility were not sufficient for clinical implementation. However, scanner-specific optimization strategies proved effective in mitigating inter-scanner variability, resulting in higher DSC and ICC values and lower SEM and SDC values compared to default or combined optimization approaches for both nicMSlesions and LST. As such, local optimization of automatic segmentation tools may provide the improvements required to implement these in the standard clinical routine.

## CRediT authorship contribution statement

**David R. van Nederpelt:** Conceptualization, Data curation, Formal analysis, Investigation, Methodology, Project administration, Resources, Software, Visualization, Writing – original draft, Writing – review & editing. **Giuseppe Pontillo:** Writing – review & editing, Methodology, Investigation, Conceptualization. **Mar Barrantes-Cepas:** Writing – review & editing, Methodology, Investigation. **Iman Brouwer:** Writing – review & editing, Software, Resources, Methodology, Investigation. **Eva M.M. Strijbis:** Writing – review & editing, Methodology, Data curation, Conceptualization. **Menno M. Schoonheim:** Writing – review & editing, Supervision. **Bastiaan Moraal:** Writing – review & editing, Data curation. **Bas Jasperse:** Writing – review & editing, Data curation. **Henk-Jan M.M. Mutsaerts:** Writing – review & editing, Supervision, Data curation. **Joep Killestein:** Writing – review & editing, Supervision, Funding acquisition, Conceptualization. **Frederik Barkhof:** Writing – review & editing, Supervision, Investigation, Funding acquisition, Conceptualization. **Joost P.A. Kuijer:** Writing – review & editing, Writing – original draft, Visualization, Supervision, Methodology, Investigation, Conceptualization. **Hugo Vrenken:** Writing – review & editing, Writing – original draft, Supervision, Methodology, Investigation, Conceptualization.

## Declaration of competing interest

The authors declare the following financial interests/personal relationships which may be considered as potential competing interests: **D.R.v.N., B.M., B.J. and J.P.A.K.** have nothing to disclose. **G.P.** has received research grants from ECTRIMS, MAGNIMS, and ESNR. **M.B.C.** is supported by research grants from Merck and Atara Biotherapeutics. **I.B.** has received research support from Merck, Novartis, Teva and the Dutch MS Research Foundation. **E.M.M.S.** received speaker fees from Merck and Novartis. **F.B.** is a steering committee or Data Safety Monitoring Board member for Biogen, Merck, Eisai and Prothena. Advisory board member for Combinostics, Scottish Brain Sciences. Consultant for Roche, Celltrion, Rewind Therapeutics, Merck, Bracco. Research agreements with ADDI, Merck, Biogen, GE Healthcare, Roche. Co-founder and shareholder of Queen Square Analytics LTD. **H.V.** has received research support from Merck, Novartis, Pfizer, and Teva; consulting fees from Merck, and speaker honoraria from Novartis; all funds were paid to his institution. **J. K.** received research grants for multicentre investigator initiated trials DOT-MS trial, ClinicalTrials. gov Identifier: NCT04260711 (ZonMW) and BLOOMS trial (ZonMW and Treatmeds), ClinicalTrials. gov Identifier: NCT05296161); received consulting fees for F. Hoffmann-La Roche, Biogen, Teva, Merck, Novartis and Sanofi/Genzyme (all payments to institution); reports speaker relationships with F. Hoffmann-La Roche, Biogen, Immunic, Teva, Merck, Novartis and Sanofi/Genzyme (all payments to institution); adjudication committee of MS clinical trial of Immunic (payments to institution only). **M.M.S.** serves on the editorial board of *Neurology* and *Frontiers in Neurology*; receives research support from the Dutch MS Research Foundation, Eurostars-EUREKA, ARSEP, Amsterdam Neuroscience, MAGNIMS, and ZonMW (Vidi grant, project number 09150172010056); and has served as a consultant for or received research support from Atara Biotherapeutics, Biogen, Celgene/Bristol Meyers Squibb, EIP, Sanofi, MedDay, and Merck. **H.J.M.M.M.** is supported by the Dutch Heart Foundation (03–004-2020-T049), by the Eurostars-2 joint programme with co-funding from the European Union Horizon 2020 research and innovation programme (ASPIRE E!113701), provided by the Netherlands Enterprise Agency (RvO), and by the EU Joint Program for Neurodegenerative Disease Research, provided by the Netherlands Organisation for health Research and Development and Alzheimer Nederland (DEBBIE JPND2020-568–106).

## Data Availability

Data will be made available on request.
