# Supplementary material for: Scanner-specific optimisation of automated lesion segmentation in MS
Source: Neuroimage Clin. 2024 Oct 2;44:103680. doi: 10.1016/j.nicl.2024.103680 (PMC11492079; doi:10.1016/j.nicl.2024.103680)
Supplement: Supplementary Data 1 [file mmc1.docx]

# Supplementary materials

Supplementary table 1: Demographics of participants used for optimalisation purposes.

| Subject | Lesion volume (mL) | Type | Age (years) | Sex | Lesion count | EDSS |
| --- | --- | --- | --- | --- | --- | --- |
| Closest to median (below) | 5.2 | RRMS | 21 | Female | 59 | 3.0 |
| Median | 6.7 | RRMS | 52 | Male | 33 | 2.5 |
| Closest to median (above) | 6.9 | RRMS | 49 | Female | 46 | 5.5 |


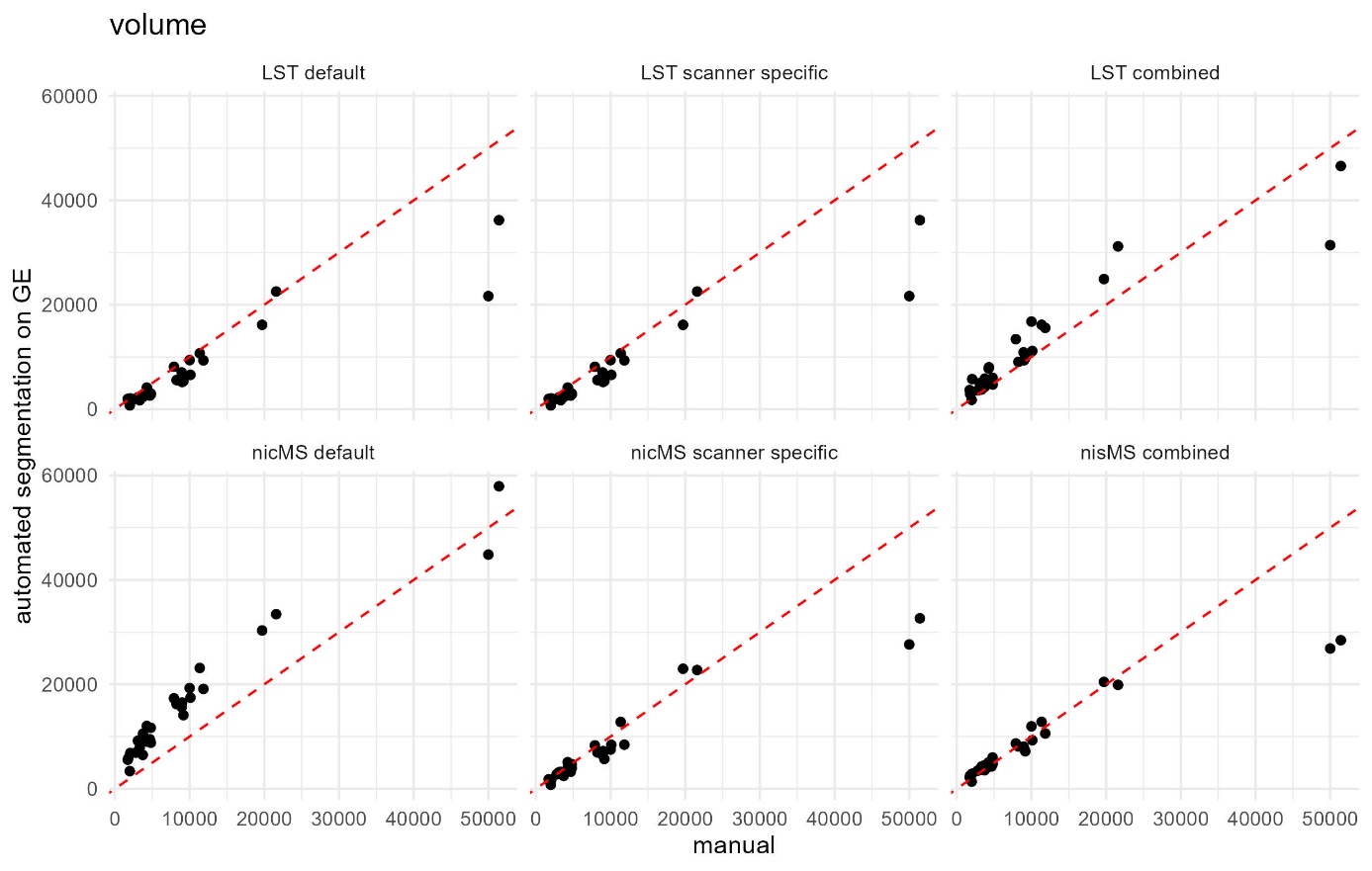


**Supplementary figure 1.** Scatter plot oflesion volumes of GE run 1 plotted against manual volumes in native FLAIR space of the GE run 1. The red dashed line is the identity line.
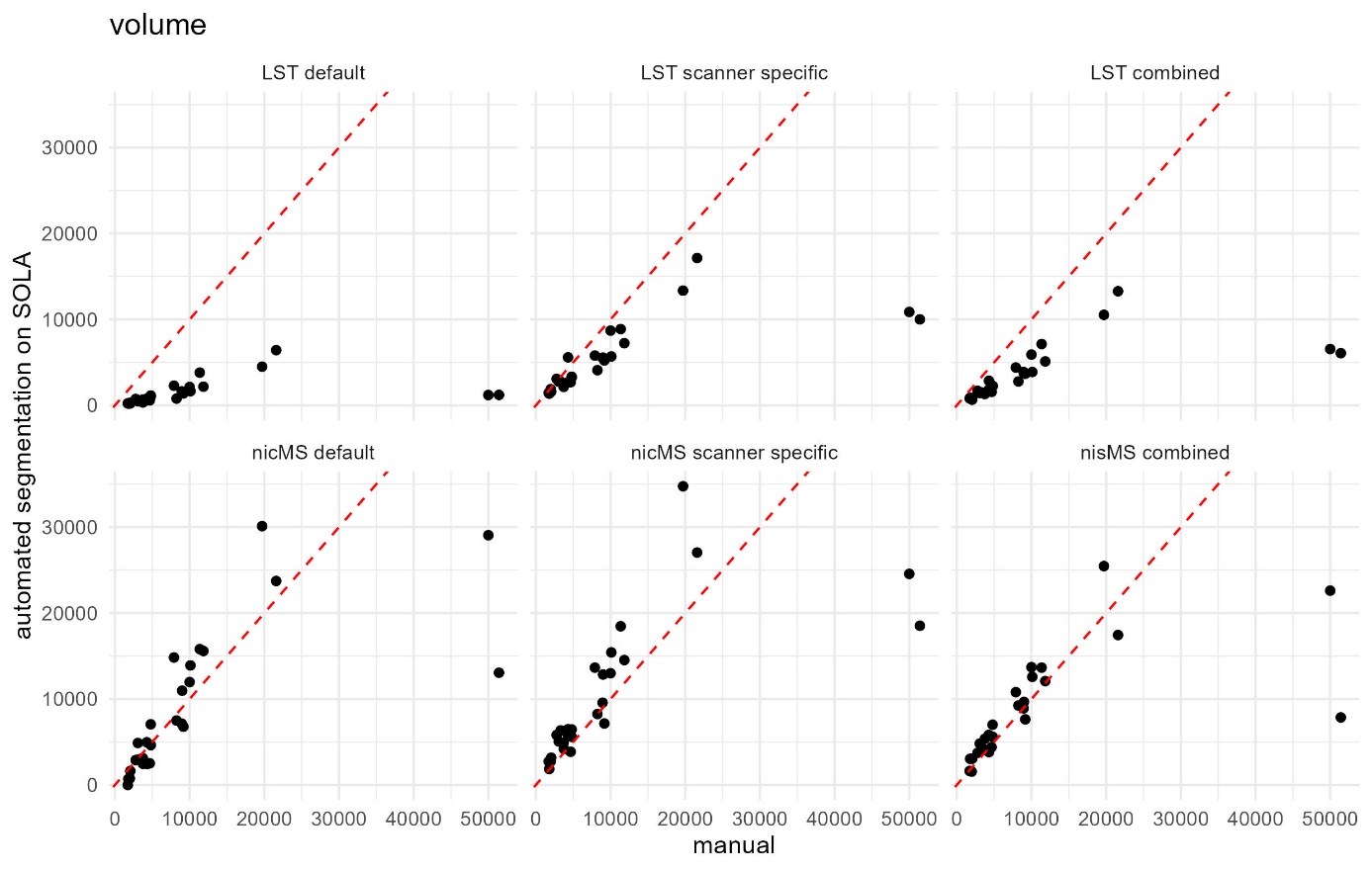


**Supplementary figure 2.** Scatter plot of lesion volumes of SOLA run 1 plotted against manual volumes in native FLAIR space of the SOLA run 1. The red dashed line is the identity line.

#
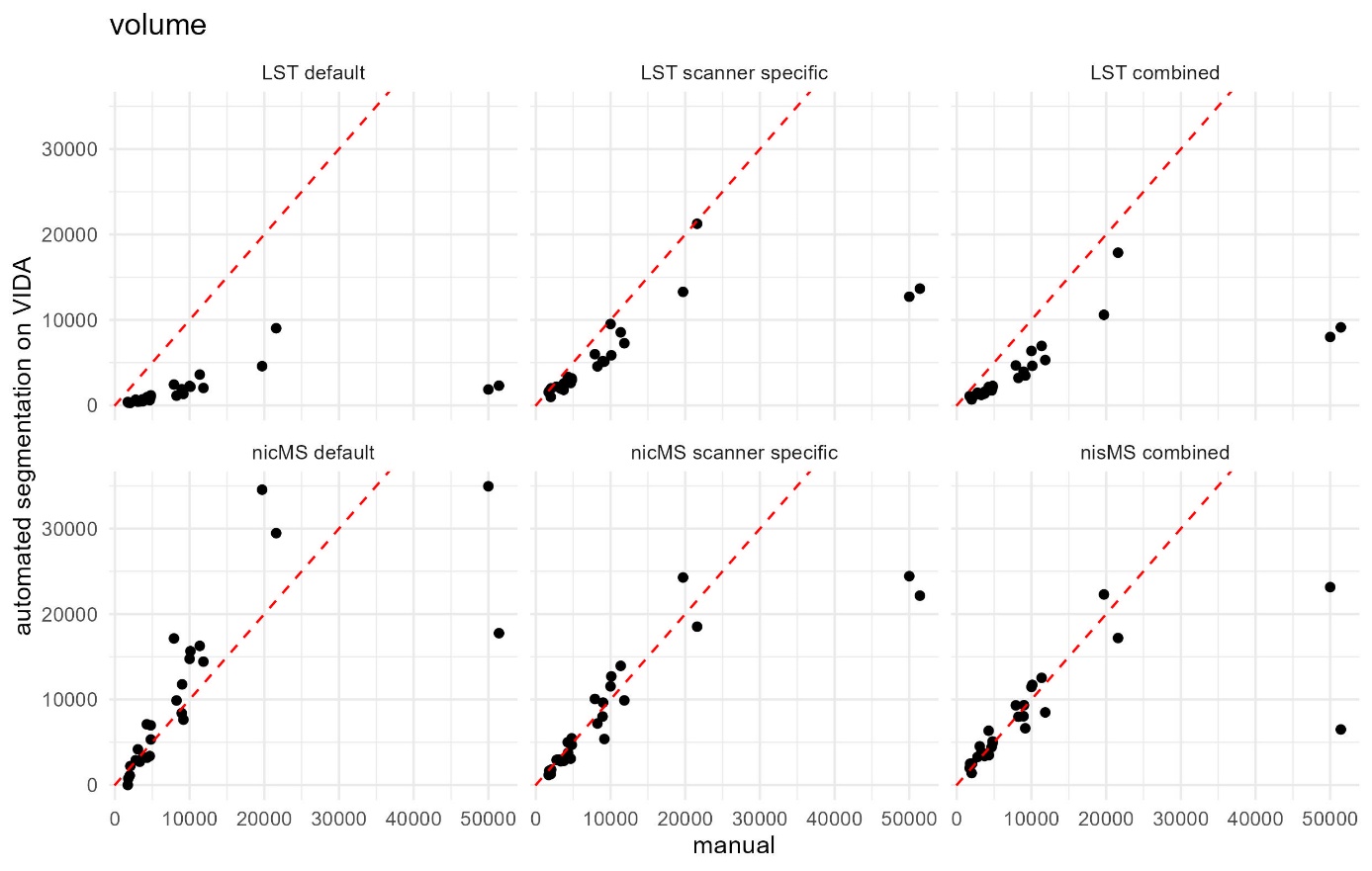


**Supplementary figure 3.** Scatter plot of lesion volumes of VIDA run 1 plotted against manual volumes in native FLAIR space of the VIDA run 1. The red dashed line is the identity line.
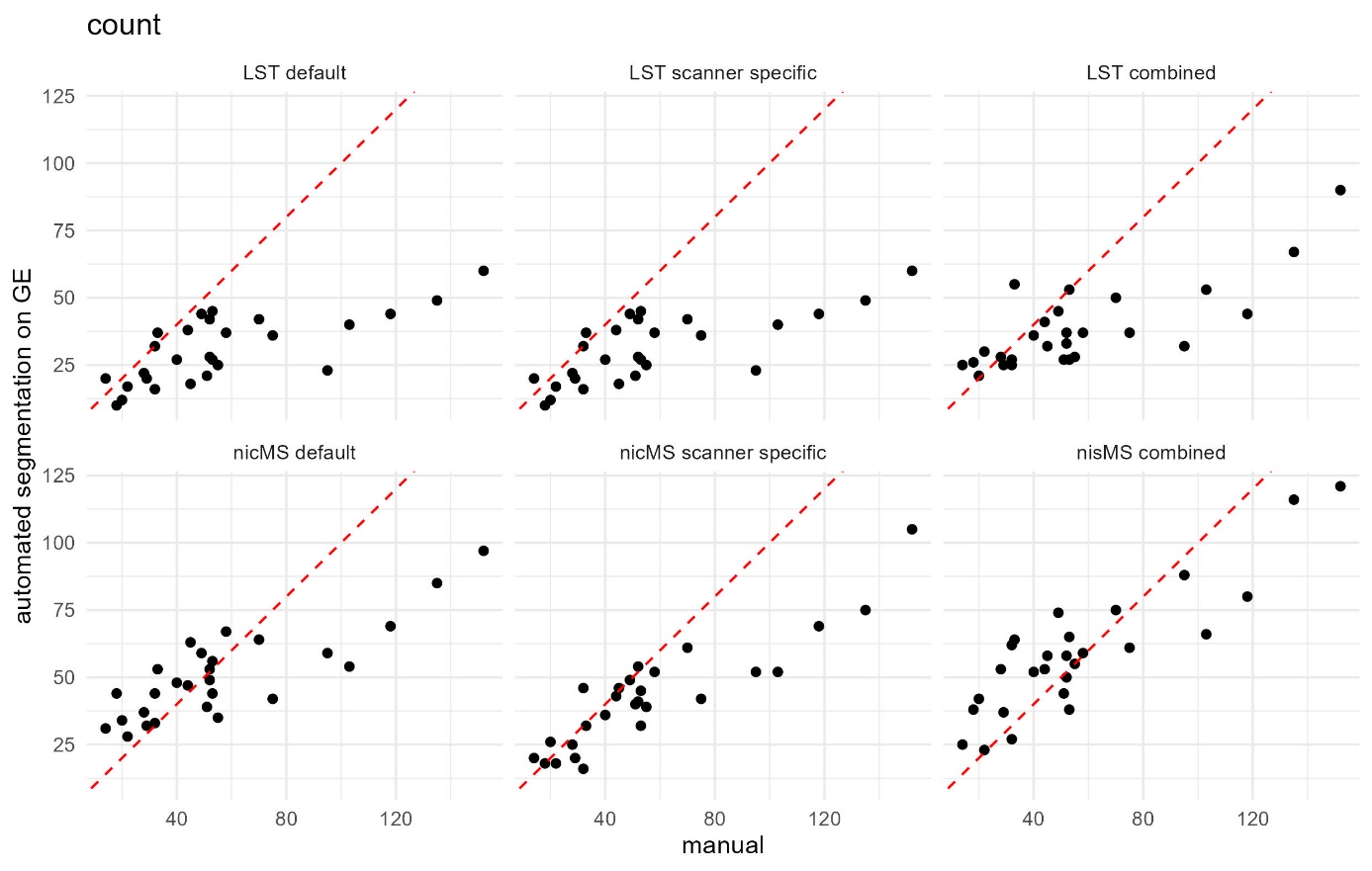


**Supplementary figure 4.** Scatter plot of lesion counts of GE run 1 plotted against manual counts in native FLAIR space of the GE run 1. The red dashed line is the identity line


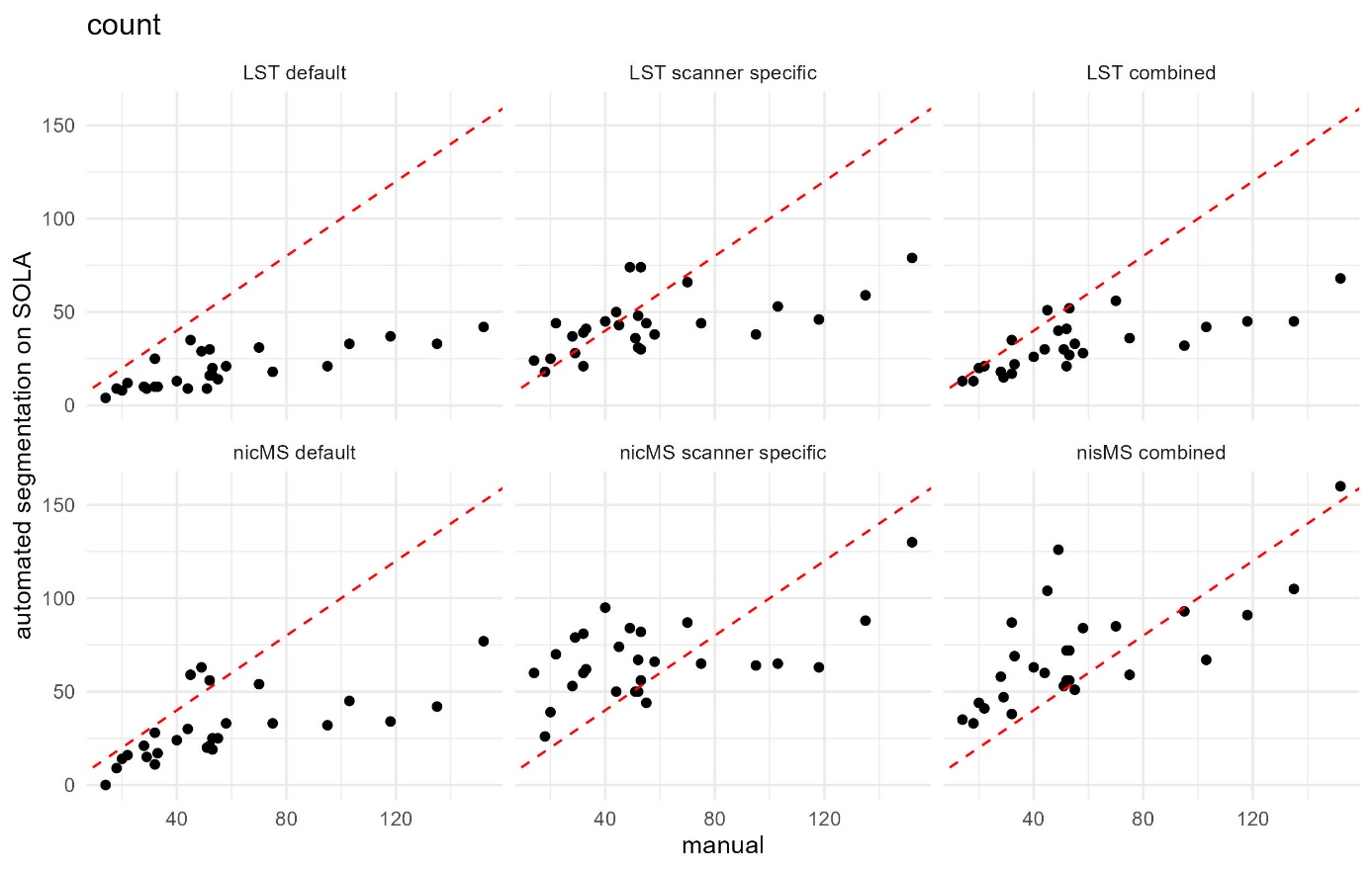


**Supplementary figure 5.** Scatter plot of lesion count of SOLA run 1 plotted against manual counts in native FLAIR space of the SOLA run 1. The red dashed line is the identity line.


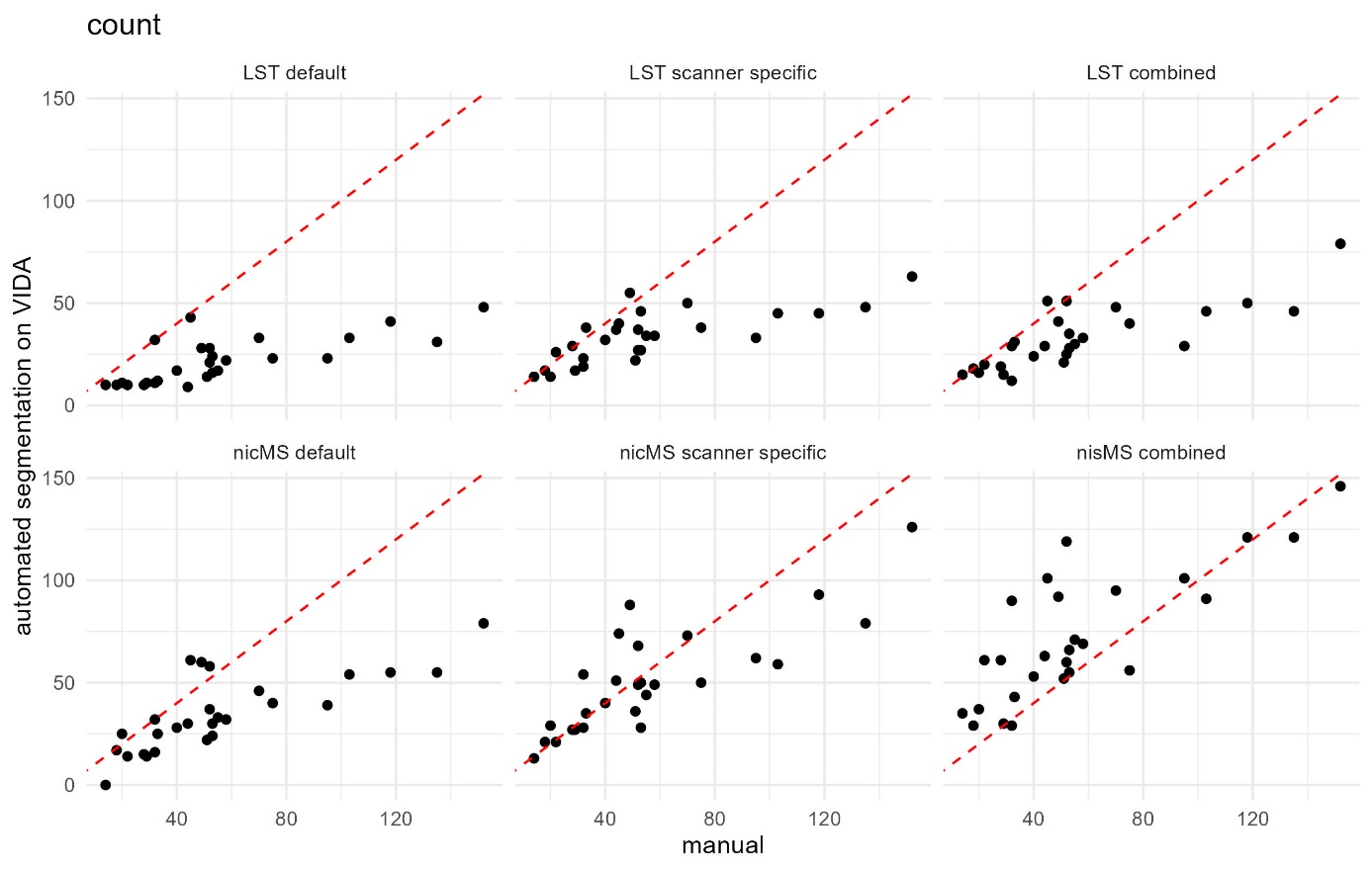


**Supplementary figure 6.** Scatter plot of lesion counts of VIDA run 1 plotted against manual counts in native FLAIR space of the VIDA run 1. The red dashed line is the identity line.


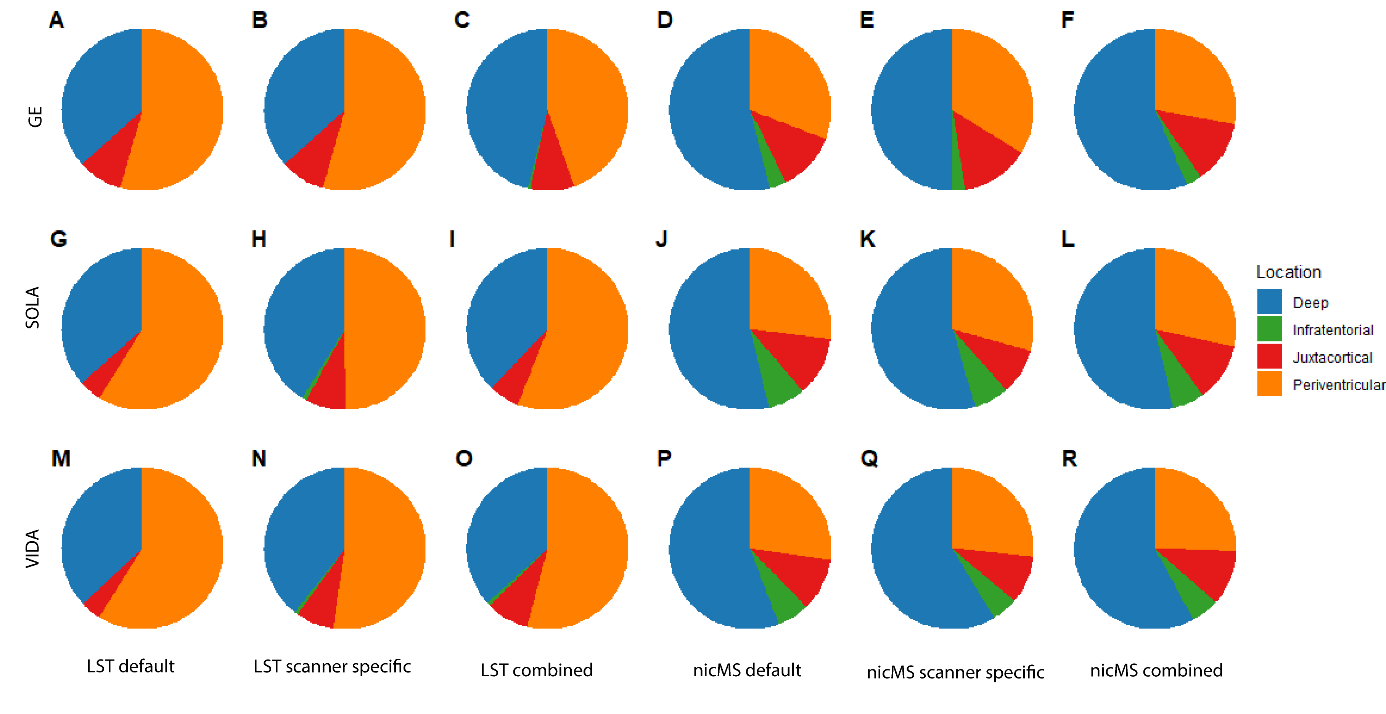


**Supplementary figure 7.** Pie chart for the percentage of locations of the true positive lesions. A = LST default on GE, B = LST scanner scanner specific on GE, C = LST combined on GE, D = nicMS default on GE, E = nicMS scanner specific on GE, F = nicMS combined on GE, G = LST default on Sola, H = LST scanner specific on Sola, I = LST comined on Sola, J = nicMS default on Sola, K = nicMS scanner specific on Sola, L = nicMS combined on Sola, M = LST default on Vida, N = LST scanner specific on Vida, O = LST combined on Vida, P = nicMS default on Vida, Q = nicMS scanner specific on Vida, R = nicMS combined on Vida.


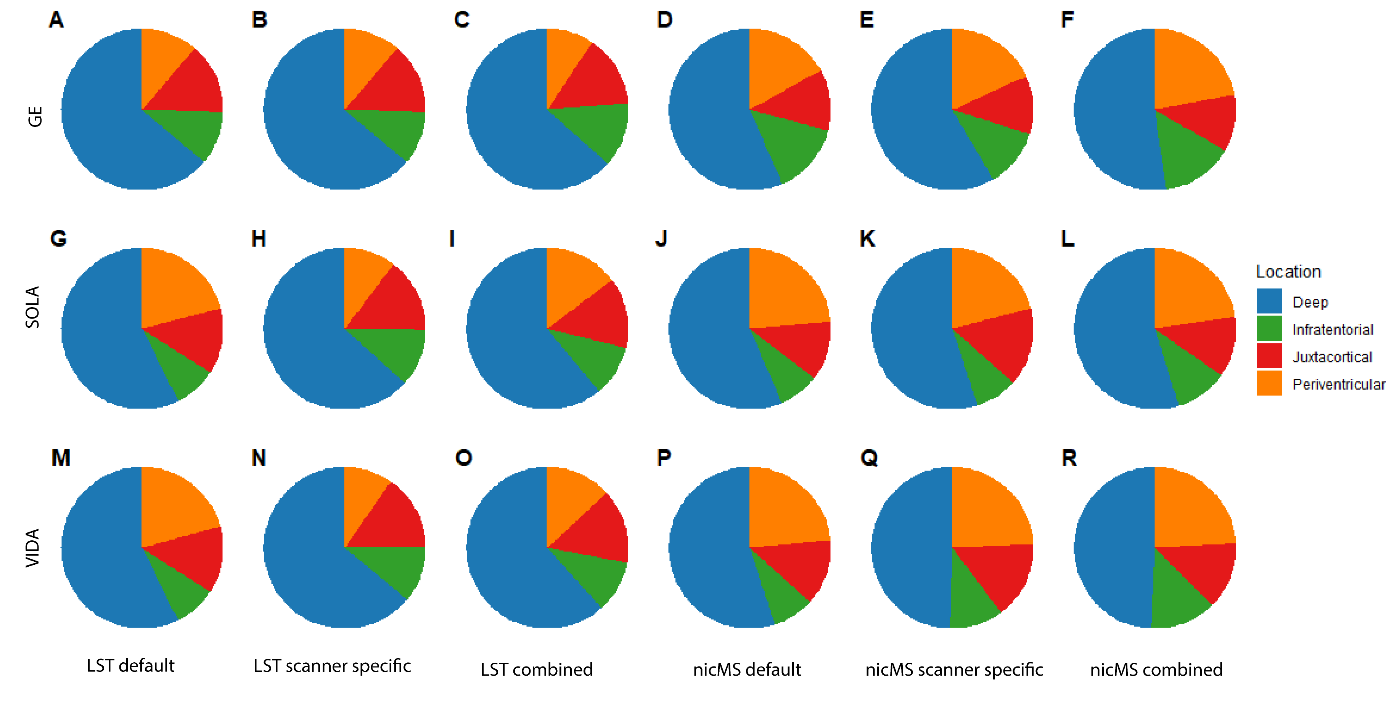


**Supplementary figure 8.** Pie chart for the percentage of locations of the false negative lesions. A = LST default on GE, B = LST scanner scanner specific on GE, C = LST combined on GE, D = nicMS default on GE, E = nicMS scanner specific on GE, F = nicMS combined on GE, G = LST default on Sola, H = LST scanner specific on Sola, I = LST comined on Sola, J = nicMS default on Sola, K = nicMS scanner specific on Sola, L = nicMS combined on Sola, M = LST default on Vida, N = LST scanner specific on Vida, O = LST combined on Vida, P = nicMS default on Vida, Q = nicMS scanner specific on Vida, R = nicMS combined on Vida.


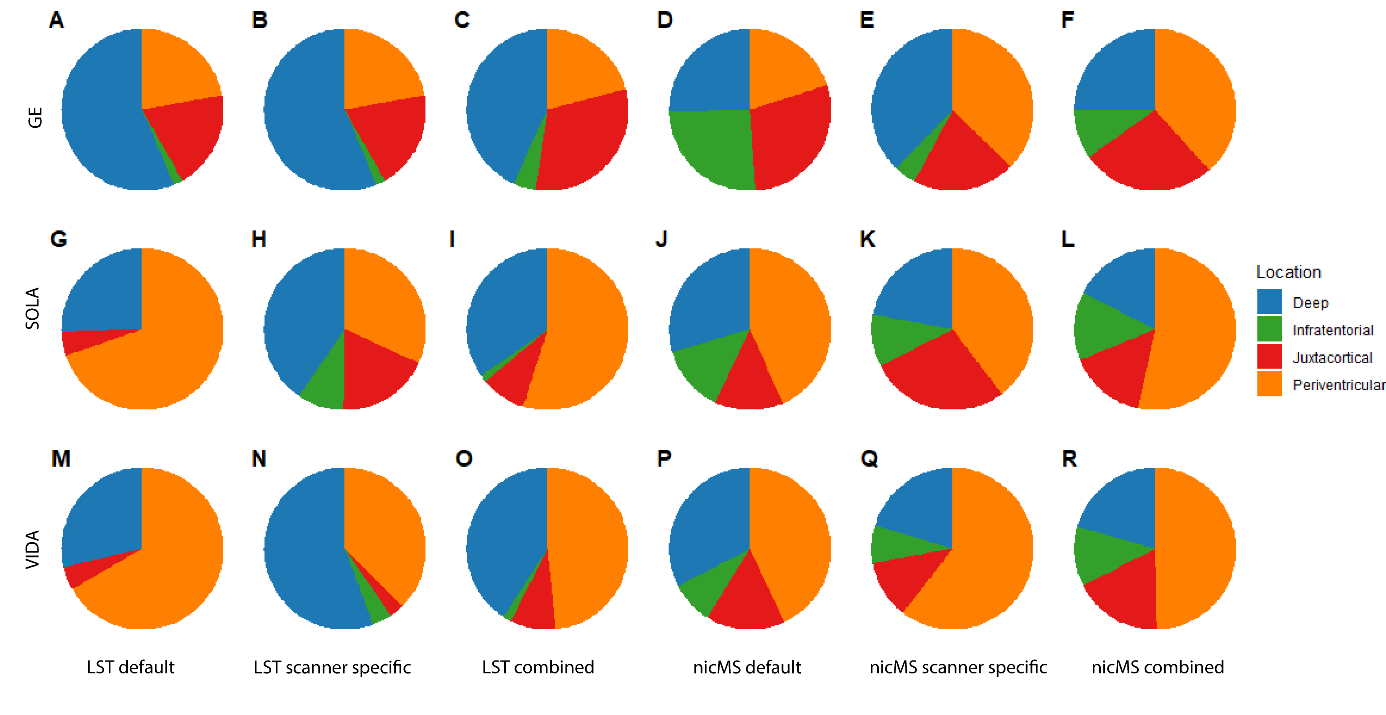


**Supplementary figure 9.** Pie chart for the percentage of locations of the false positive lesions. A = LST default on GE, B = LST scanner scanner specific on GE, C = LST combined on GE, D = nicMS default on GE, E = nicMS scanner specific on GE, F = nicMS combined on GE, G = LST default on Sola, H = LST scanner specific on Sola, I = LST comined on Sola, J = nicMS default on Sola, K = nicMS scanner specific on Sola, L = nicMS combined on Sola, M = LST default on Vida, N = LST scanner specific on Vida, O = LST combined on Vida, P = nicMS default on Vida, Q = nicMS scanner specific on Vida, R = nicMS combined on Vida.


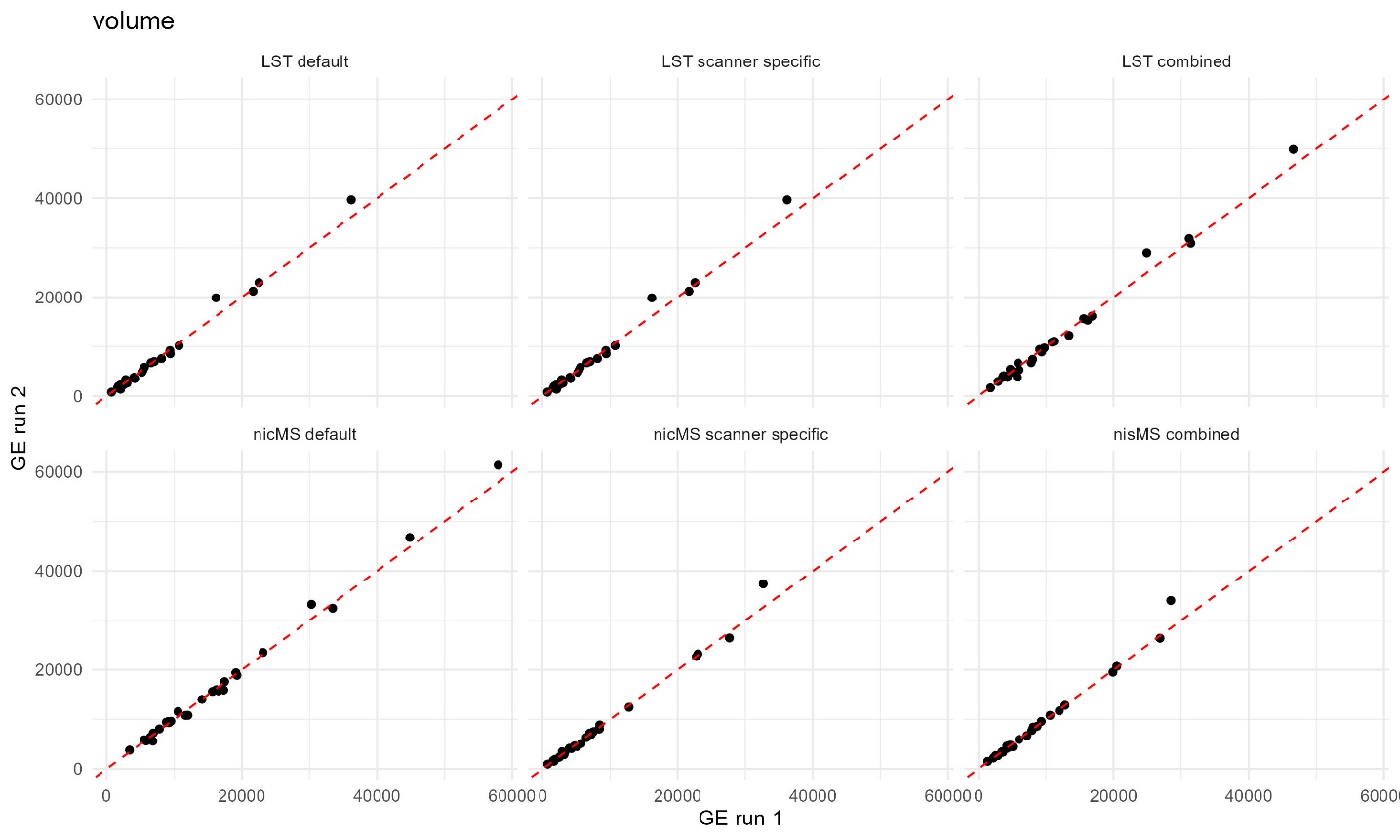


**Supplementary figure 10.** Lesion volumes of GE run 1 plotted against GE run 2. The red dashed line is the identity line.


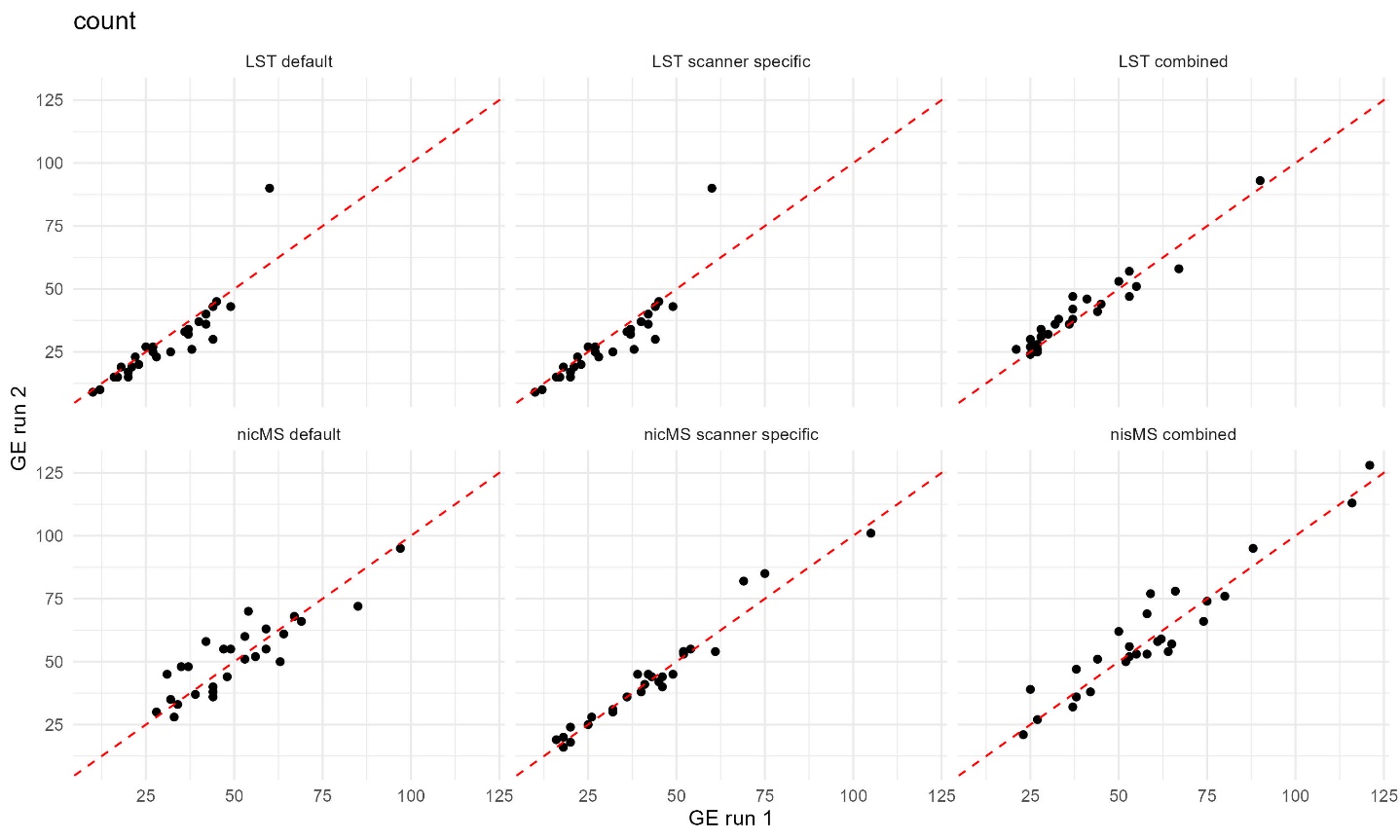


**Supplementary figure 11.** Lesion count of GE run 1 plotted against GE run 2. The red dashed line is the identity line.


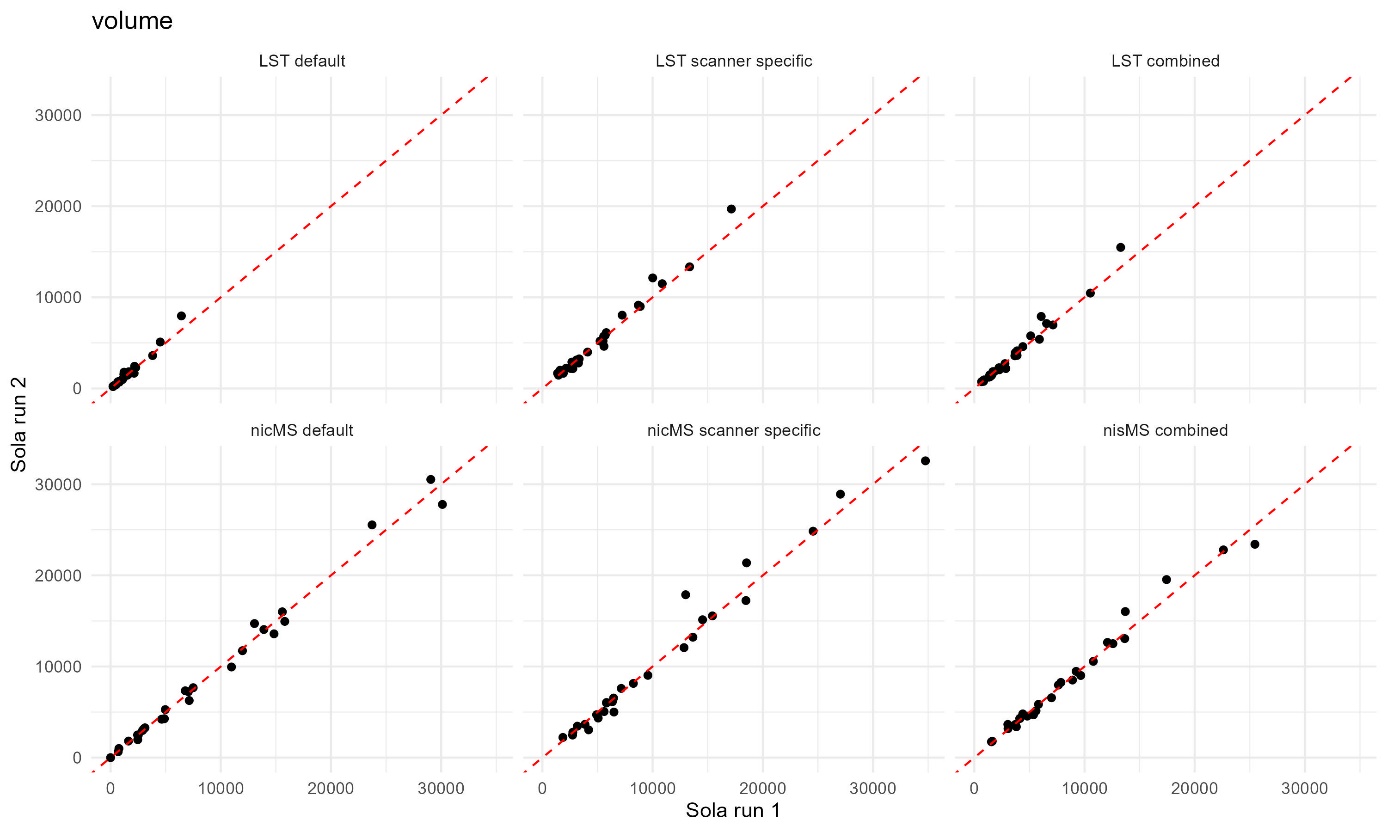


**Supplementary figure 12.** Lesion volumes of Sola run 1 plotted against Sola run 2. The red dashed line is the identity line.


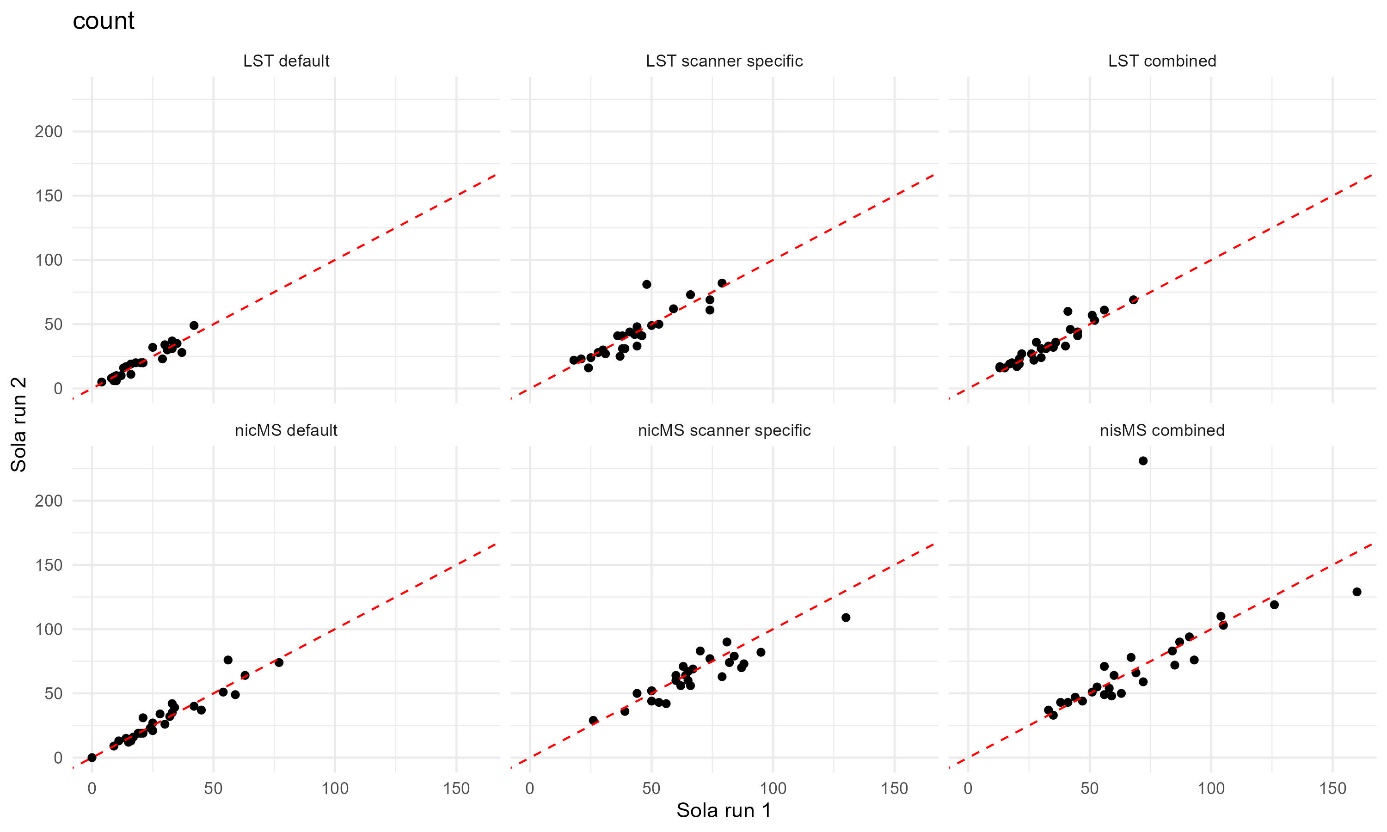


**Supplementary figure 13.** Lesion count of Sola run 1 plotted against Sola run 2. The red dashed line is the identity line.


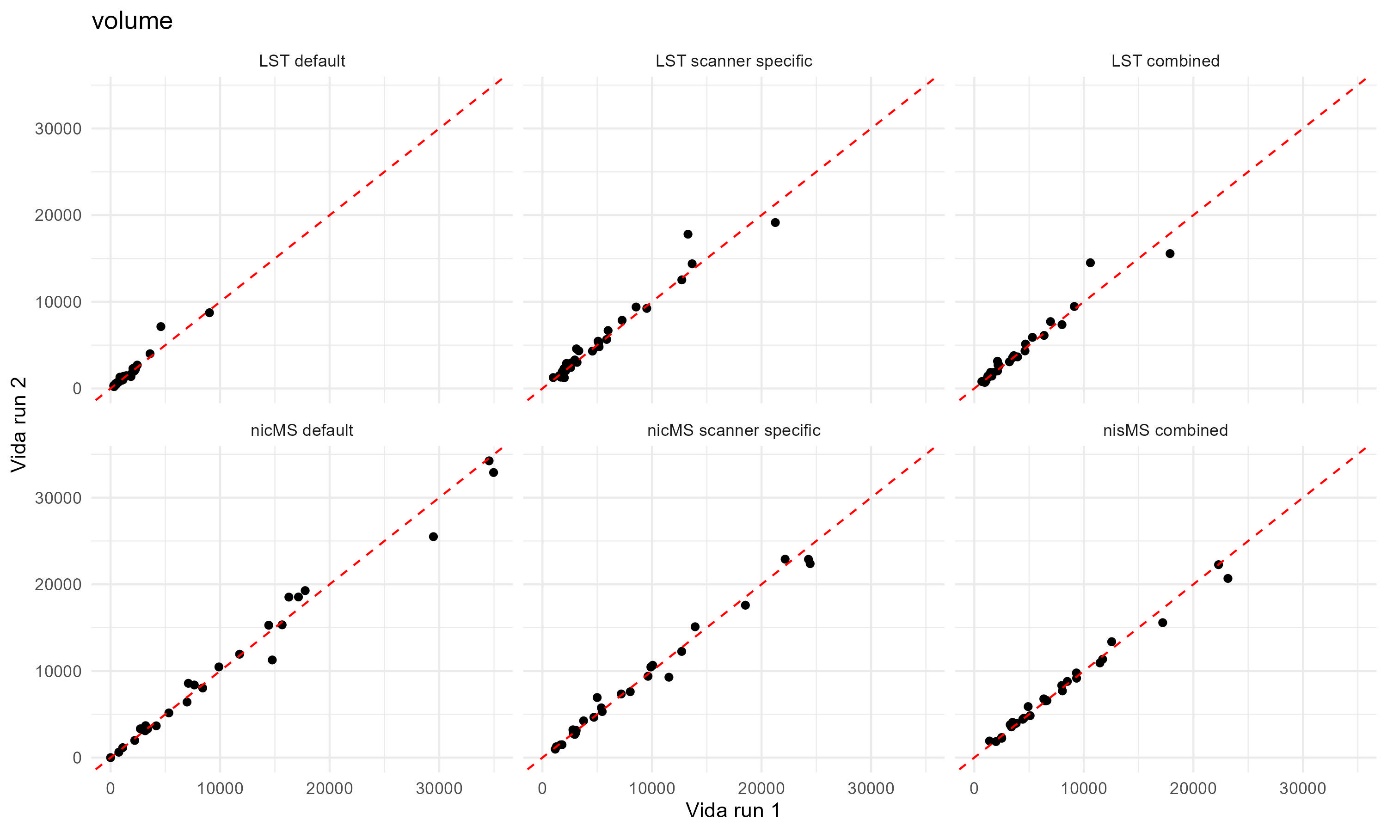


**Supplementary figure 14.** Lesion volumes of Vida run 1 plotted against Vida run 2. The red dashed line is the identity line.


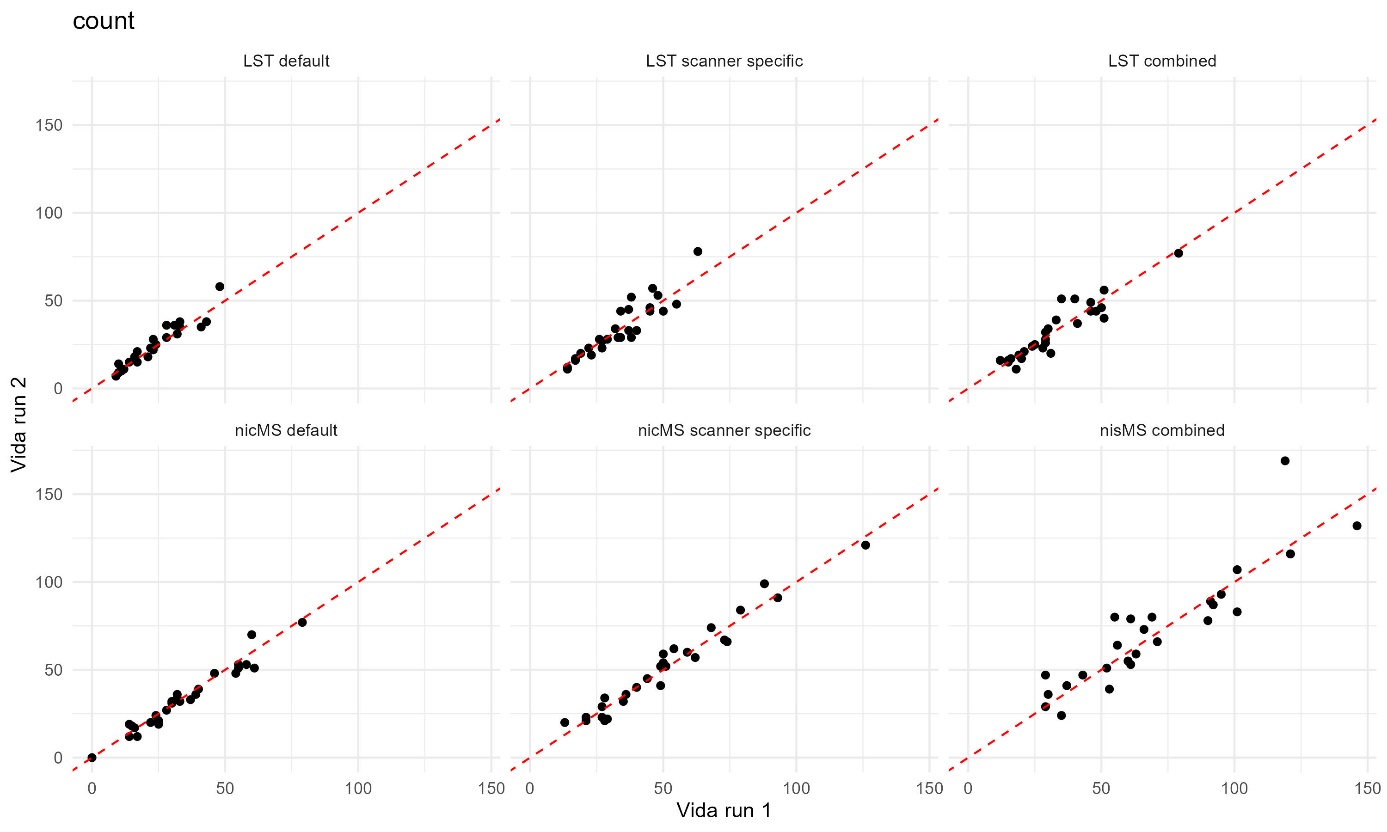


**Supplementary figure 15.** Lesion count of Vida run 1 plotted against Vida run 2. The red dashed line is the identity line.


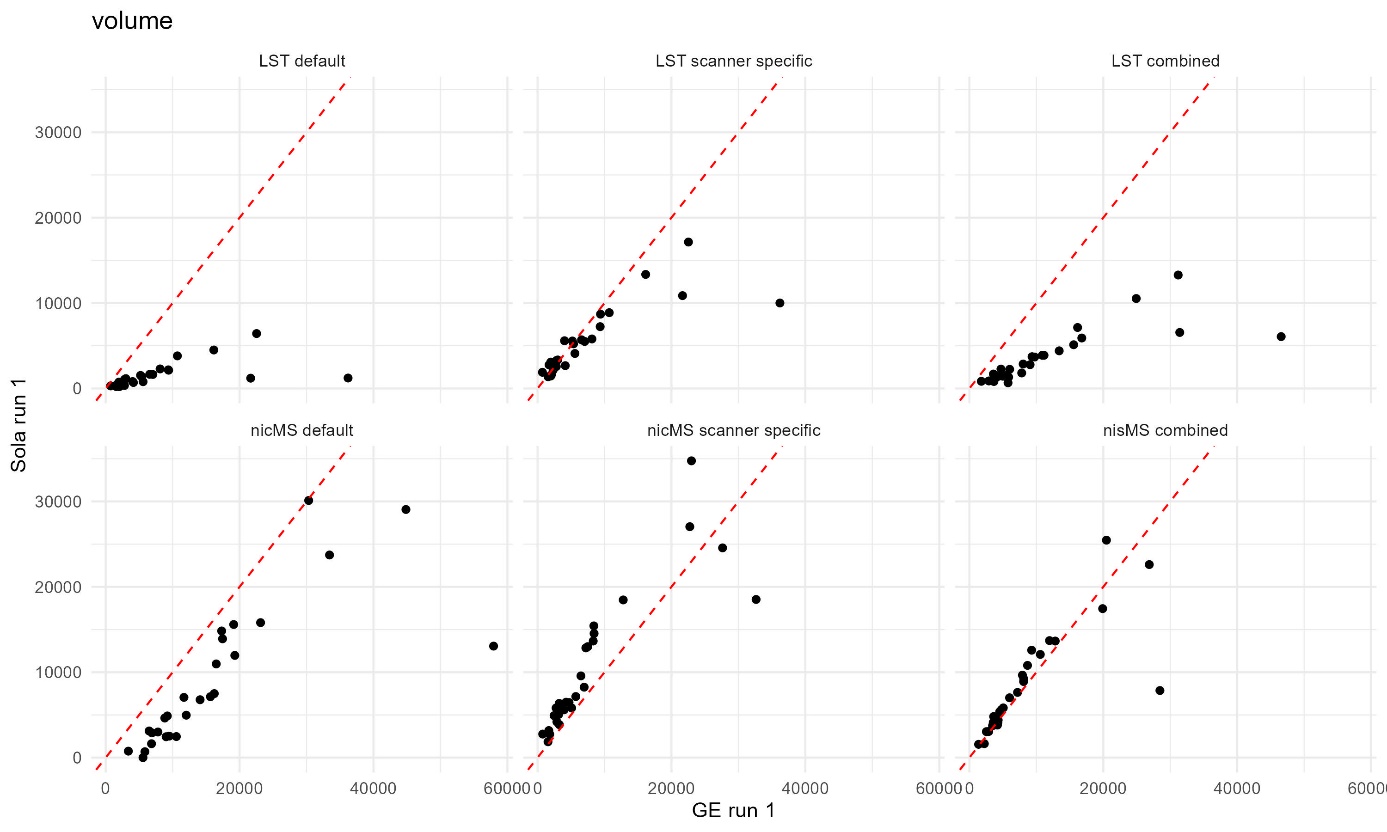


**Supplementary figure 16.** Lesion volumes of GE run 1 plotted against Sola run 1. The red dashed line is the identity line.


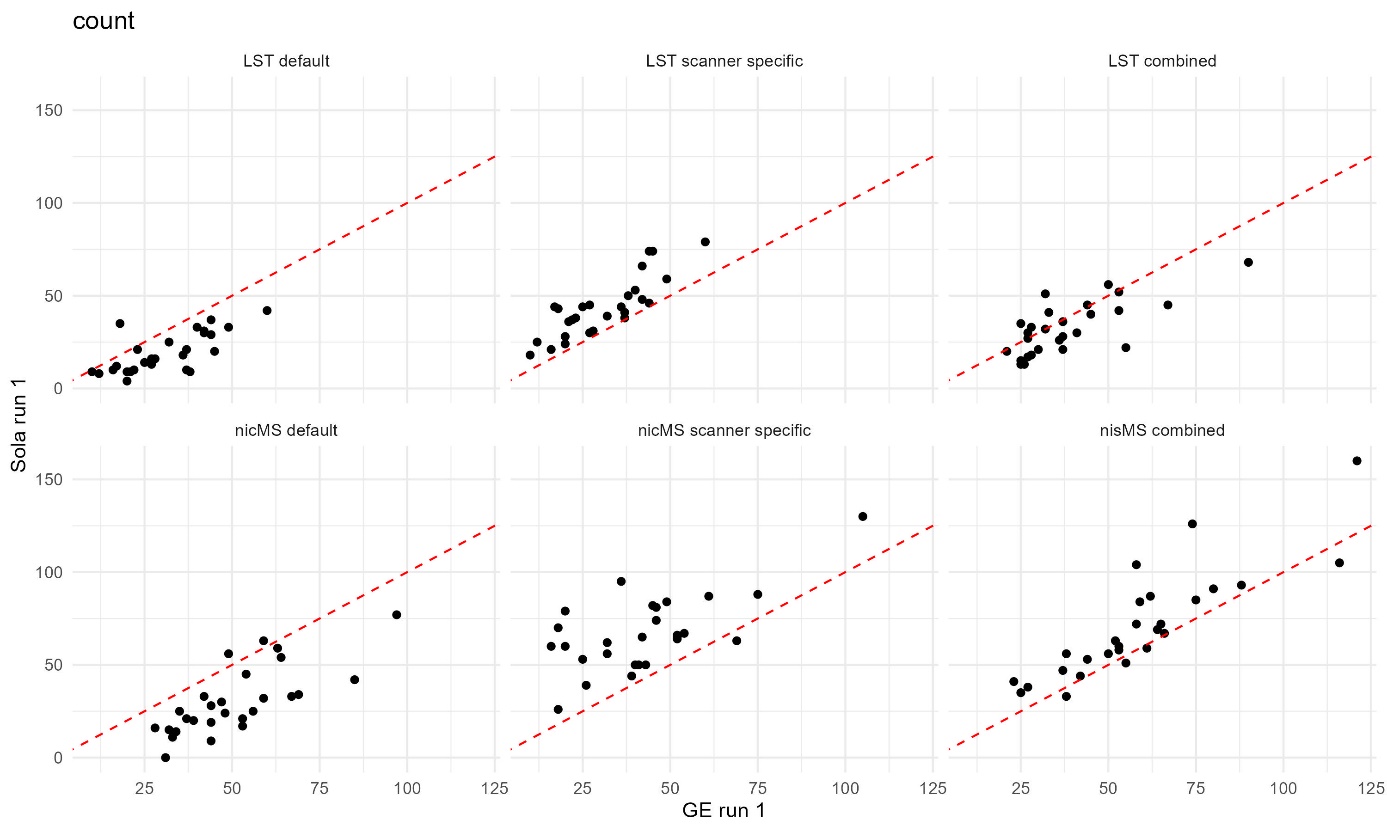


**Supplementary figure 17.** Lesion count of GE run 1 plotted against Sola run 1. The red dashed line is the identity line.


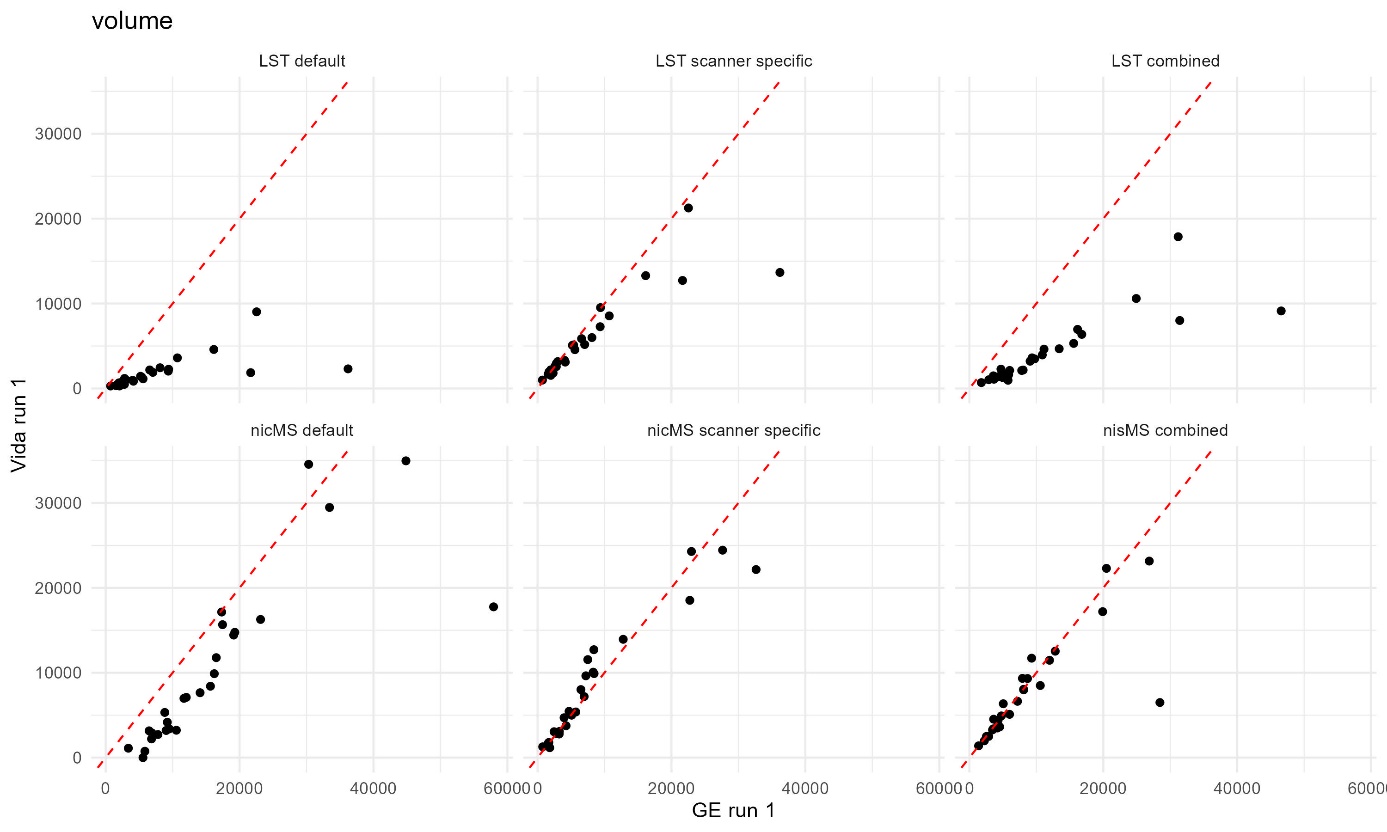


**Supplementary figure 18.** Lesion volumes of GE run 1 plotted against Vida run 1. The red dashed line is the identity line.


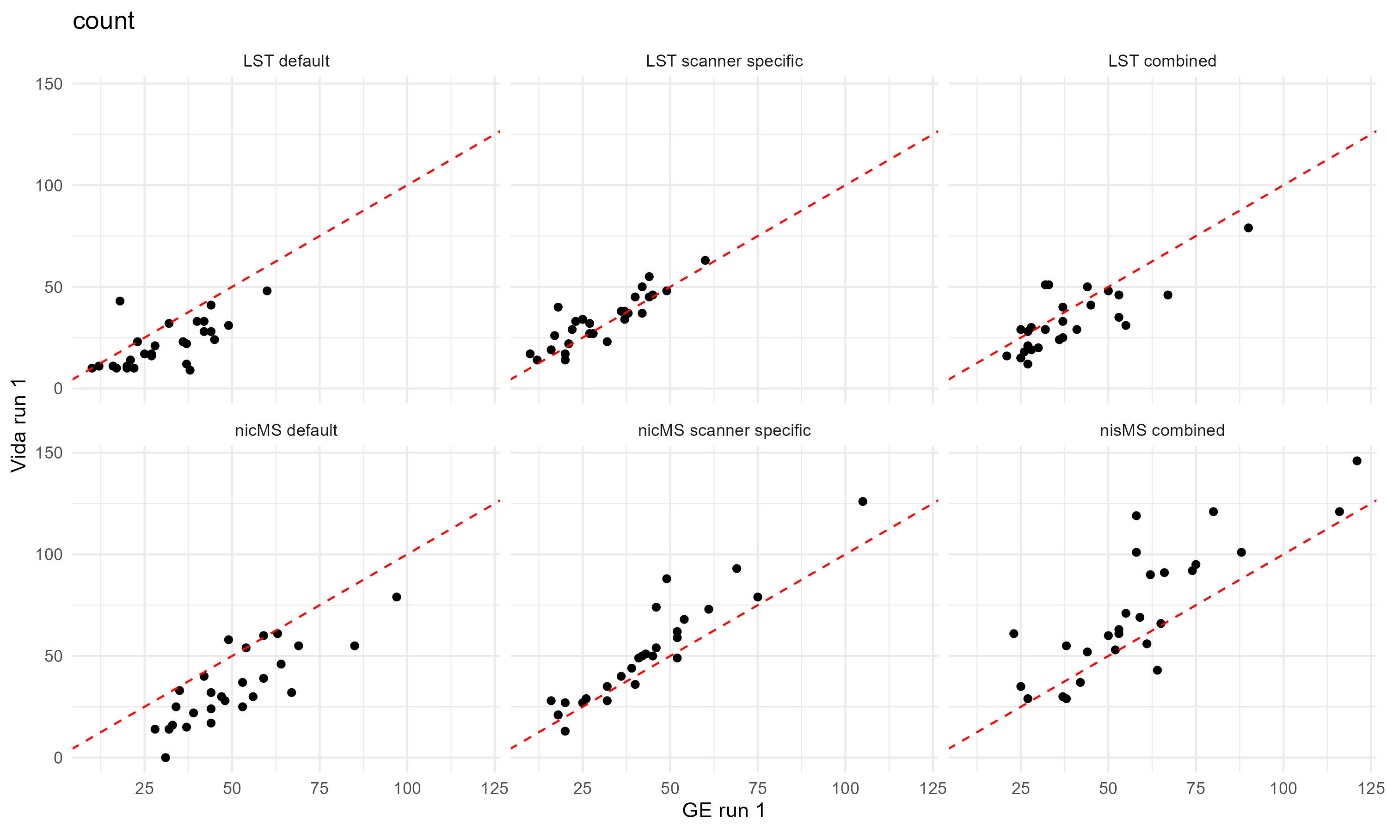


**Supplementary figure 19.** Count of GE run 1 plotted against Vida run 1. The red dashed line is the identity line.


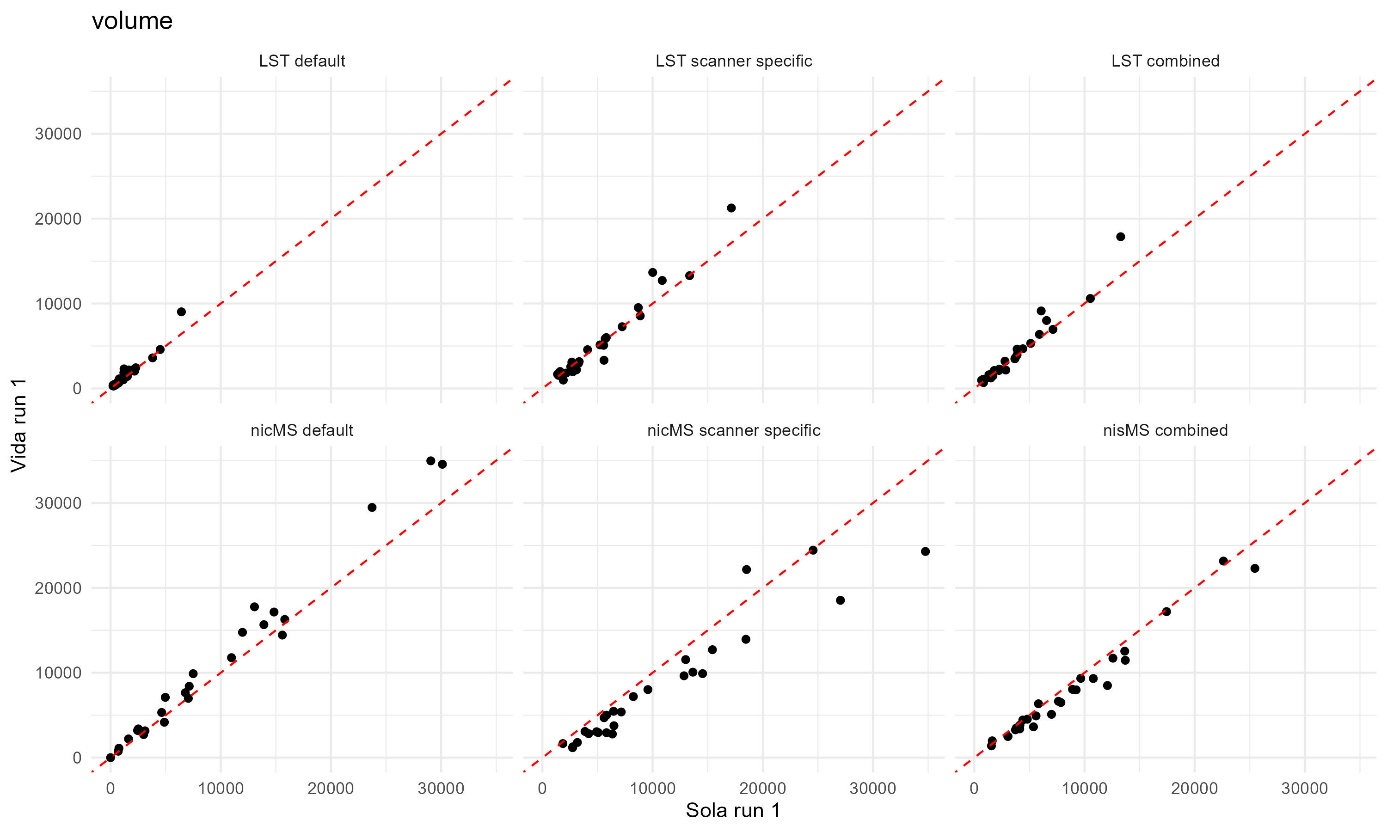


**Supplementary figure 20.** Lesion volumes of Sola run 1 plotted against Vida run 1. The red dashed line is the identity line.


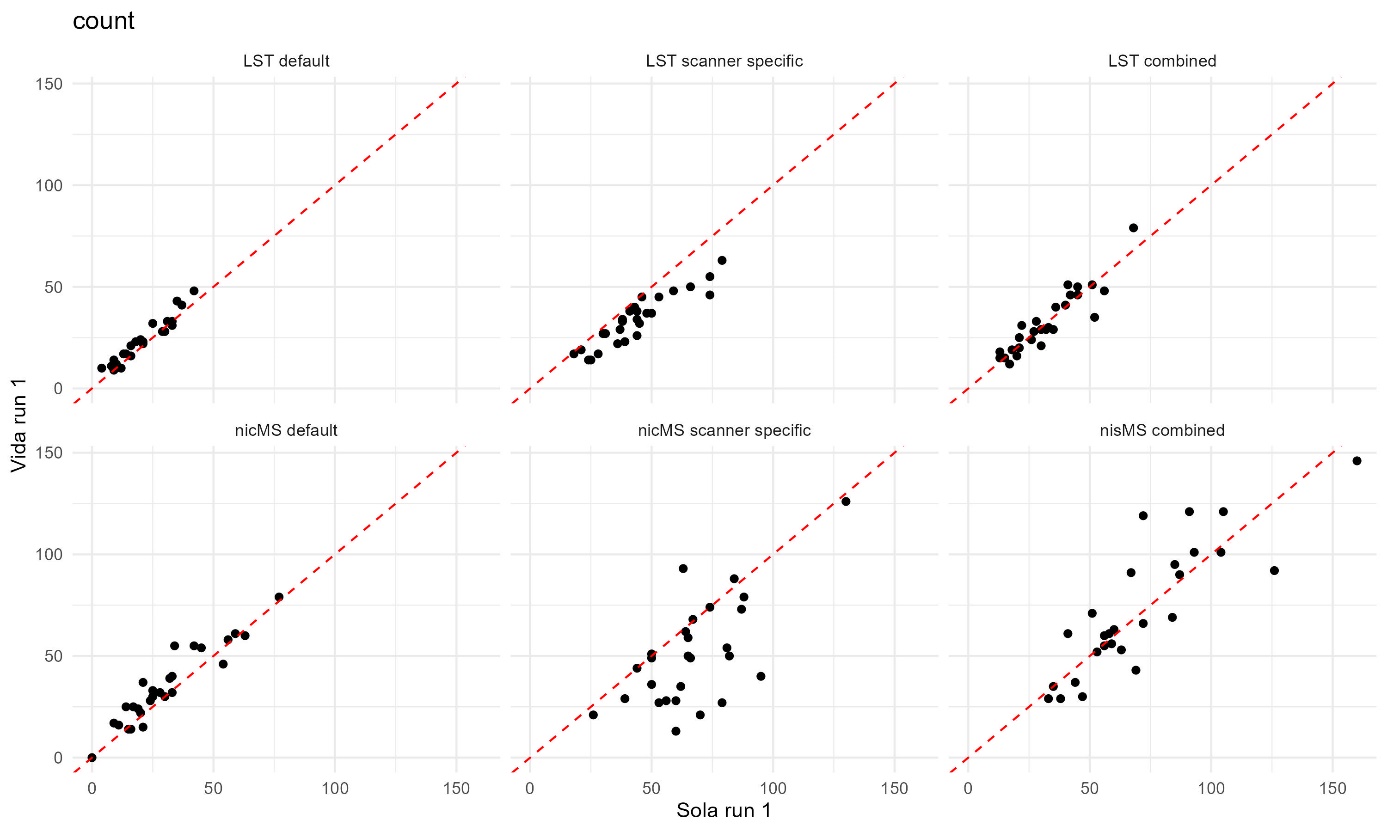


**Supplementary figure 21.** Count of Sola run 1 plotted against Vida run 1. The red dashed line is the identity line.


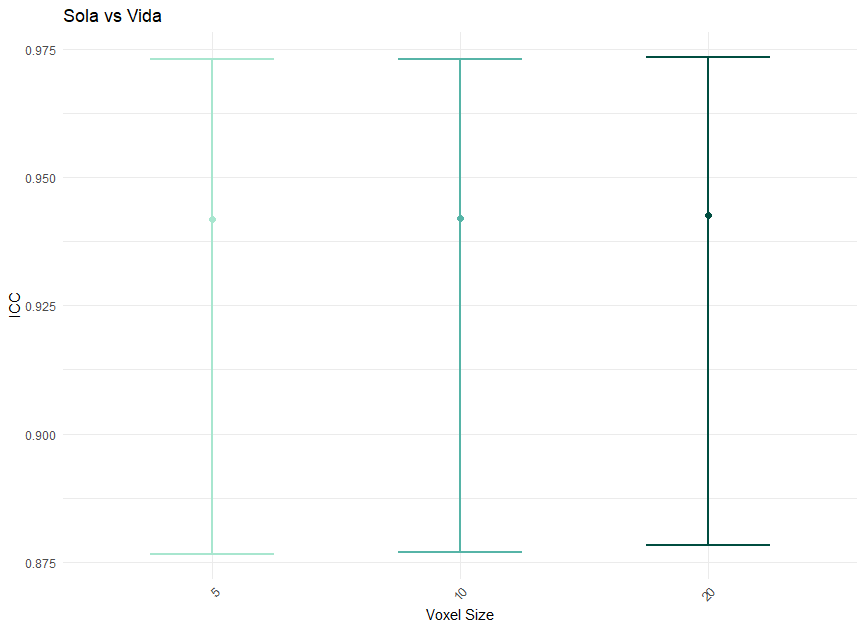


**Supplementary Figure 22.** The Intra-class correlation coefficient for consistency between scanners Sola and Vida for different lesion cluster sizes of 5, 10 and 20 voxels on lesion volume. The error bars are the 95% confidence intervals.
